# Australia: A Continent Without Native Powdery Mildews? The First Comprehensive Catalog Indicates Recent Introductions and Multiple Host Range Expansion Events, and Leads to the Re-discovery of *Salmonomyces* as a New Lineage of the Erysiphales

**DOI:** 10.3389/fmicb.2020.01571

**Published:** 2020-07-16

**Authors:** Levente Kiss, Niloofar Vaghefi, Kaylene Bransgrove, John D. W. Dearnaley, Susumu Takamatsu, Yu Pei Tan, Craig Marston, Shu-Yan Liu, Dan-Ni Jin, Dante L. Adorada, Jordan Bailey, Maria Graciela Cabrera de Álvarez, Andrew Daly, Pamela Maia Dirchwolf, Lynne Jones, Thuan Dat Nguyen, Jacqueline Edwards, Wellcome Ho, Lisa Kelly, Sharl J. L. Mintoff, Jennifer Morrison, Márk Z. Németh, Sandy Perkins, Roger G. Shivas, Reannon Smith, Kara Stuart, Ronald Southwell, Unaisi Turaganivalu, Kálmán Zoltán Váczy, Annie Van Blommestein, Dominie Wright, Anthony Young, Uwe Braun

**Affiliations:** ^1^Centre for Crop Health, Institute for Life Sciences and the Environment, University of Southern Queensland, Toowoomba, QLD, Australia; ^2^Queensland Plant Pathology Herbarium, Department of Agriculture and Fisheries, Dutton Park, QLD, Australia; ^3^Laboratory of Plant Pathology, Faculty of Bioresources, Mie University, Tsu, Japan; ^4^Science and Surveillance Group, Department of Agriculture, Water and the Environment, Brisbane, QLD, Australia; ^5^College of Plant Protection, Jilin Agricultural University, Changchun, China; ^6^Plant Pathology & Mycology Herbarium, New South Wales Department of Primary Industries, Orange, NSW, Australia; ^7^Department of Plant Protection, Faculty of Agricultural Science, National University of the Northeast, Corrientes, Argentina; ^8^Plant Health Diagnostic Service, New South Wales Department of Primary Industries, Elizabeth Macarthur Agricultural Institute, Menangle, NSW, Australia; ^9^Field Crops Research Institute, Hanoi, Vietnam; ^10^Agriculture Victoria Research, Agriculture Victoria, Department of Jobs, Precincts and Regions, Bundoora, VIC, Australia; ^11^School of Applied Systems Biology, La Trobe University, Bundoora, VIC, Australia; ^12^New Zealand Ministry for Primary Industries, Auckland, New Zealand; ^13^Department of Agriculture and Fisheries, Queensland Government, Toowoomba, QLD, Australia; ^14^Department of Primary Industry and Resources, Northern Territory Government, Darwin, NT, Australia; ^15^Plant Protection Institute, Centre for Agricultural Research, Budapest, Hungary; ^16^Ecosciences Precinct, Department of Agriculture and Fisheries, Dutton Park, QLD, Australia; ^17^Science and Surveillance Group, Department of Agriculture, Water and the Environment, Sydney, NSW, Australia; ^18^Secretariat for the Pacific Community, Suva, Fiji; ^19^Food and Wine Research Institute, Eszterházy Károly University, Eger, Hungary; ^20^Department of Primary Industries and Regional Development, South Perth, WA, Australia; ^21^School of Agriculture and Food Sciences, The University of Queensland, Brisbane, QLD, Australia; ^22^Herbarium, Department of Geobotany and Botanical Garden, Institute for Biology, Martin Luther University, Halle (Saale), Germany

**Keywords:** host jumps, host-pathogen interactions, invasive species, obligate biotrophs, plant-microbe interactions, rapid evolution

## Abstract

In contrast to Eurasia and North America, powdery mildews (Ascomycota, Erysiphales) are understudied in Australia. There are over 900 species known globally, with fewer than currently 60 recorded from Australia. Some of the Australian records are doubtful as the identifications were presumptive, being based on host plant-pathogen lists from overseas. The goal of this study was to provide the first comprehensive catalog of all powdery mildew species present in Australia. The project resulted in (i) an up-to-date list of all the taxa that have been identified in Australia based on published DNA barcode sequences prior to this study; (ii) the precise identification of 117 specimens freshly collected from across the country; and (iii) the precise identification of 30 herbarium specimens collected between 1975 and 2013. This study confirmed 42 species representing 10 genera, including two genera and 13 species recorded for the first time in Australia. In Eurasia and North America, the number of powdery mildew species is much higher. Phylogenetic analyses of powdery mildews collected from *Acalypha* spp. resulted in the transfer of *Erysiphe acalyphae* to *Salmonomyces*, a resurrected genus. *Salmonomyces acalyphae* comb. nov. represents a newly discovered lineage of the Erysiphales. Another taxonomic change is the transfer of *Oidium ixodiae* to *Golovinomyces*. Powdery mildew infections have been confirmed on 13 native Australian plant species in the genera *Acacia, Acalypha, Cephalotus, Convolvulus, Eucalyptus, Hardenbergia, Ixodia, Jagera, Senecio*, and *Trema*. Most of the causal agents were polyphagous species that infect many other host plants both overseas and in Australia. All powdery mildews infecting native plants in Australia were phylogenetically closely related to species known overseas. The data indicate that Australia is a continent without native powdery mildews, and most, if not all, species have been introduced since the European colonization of the continent.

## Introduction

Powdery mildews (Ascomycota, Erysiphales) are common obligate biotrophic fungal plant pathogens, comprising ~900 species that infect more than 10,000 dicot and monocot plant species globally (Braun and Cook, 2012). Many powdery mildew species cause economically important diseases of agricultural and horticultural crops, including wheat, barley, grapevine, fruit, and vegetable species (Glawe, 2008). Others are forest pathogens (Marçais and Desprez-Loustau, 2014). Some powdery mildews have become invasive in different parts of the world (Kiss, 2005; Desprez-Loustau et al., 2010), posing plant health biosecurity risks (Jones and Baker, 2007; Brasier, 2008; Biosecurity Australia, 2010; Desprez-Loustau et al., 2010). Two species, *Blumeria graminis* infecting cereals, and *Erysiphe necator* infecting grapevine, have become model species in plant pathology research (Gadoury et al., 2012; Bindschedler et al., 2016), while the interactions between *Podosphaera plantaginis* and its host *Plantago lanceolata* have long been in focus in the study of wild plant pathosystems (Susi et al., 2015).

Since the early 2000s, molecular phylogenetic analyses have shown that the traditional generic concept of the Erysiphales does not mirror their phylogeny based on the morphological characteristics of the sexual morphs (chasmothecia) (Braun et al., 2002). Surprisingly, grouping species according to the characteristics of their asexual morphs reflect their molecular phylogeny (for reviews, see Takamatsu, 2004, 2013a). This discovery triggered major taxonomic revisions (Braun et al., 2002), and the currently accepted generic concept is now based on a combination of the morphological characteristics of the asexual and sexual morphs (Braun, 2011; Takamatsu, 2013b). The most recent monograph of powdery mildews recognized 17 genera (Braun and Cook, 2012). A further genus, *Bulbomicroidium*, was described from Mexico (Marmolejo et al., 2018).

In Australia, despite their importance, the biodiversity of the Erysiphales has received little attention. Based on morphological observations and field data accumulated until the early 1980s, Walker (1983) noted that powdery mildews were not recorded on native Australian plants under natural conditions. Braun and Cook (2012) listed ~50 powdery mildew species for Australia. However, some of these records are doubtful as the original identifications were presumptive, being based only on the morphology of the asexual morph, in the absence of the sexual morphs, and/or plant disease lists from overseas. It is difficult, and sometimes impossible, to distinguish powdery mildew species belonging to the same genus by morphological characteristics of the asexual morph (Takamatsu et al., 2002; Jankovics et al., 2008; Bereczky et al., 2015; Desprez-Loustau et al., 2018).

Like in other fungal groups, the advent of molecular biology has facilitated a vastly more precise dissection of the relationships among taxa. DNA barcodes, particularly sequences of the internal transcribed spacer (ITS) region of the nuclear ribosomal DNA (nrDNA), have been most useful for the identification of powdery mildews at the species level, while sequences of the nrDNA 18S and 28S region have supported the identification and classification of the genera (Braun et al., 2002; Takamatsu, 2004, 2013a; Takamatsu et al., 2015a,b; Cabrera et al., 2018; Marmolejo et al., 2018). Other DNA regions have also been tested as molecular barcodes for some groups of the powdery mildews (Inuma et al., 2007; Desprez-Loustau et al., 2017; Ellingham et al., 2019; Qiu et al., 2020), but these studies have not yet resulted in the development of new DNA barcodes that are useful to distinguish species across the Erysiphales. To date, ITS sequences is still the sole species-level DNA barcode available for this fungal group, despite its limitations. These include intragenomic variations within some powdery mildew species on one hand (Kovács et al., 2011), and no differences between some other, morphologically distinguishable species on the other hand (Braun et al., 2019; Qiu et al., 2020). ITS sequences as species barcodes have limitations in other fungal groups, as well (Kiss, 2012; Stadler et al., 2020).

Most of the species listed by Braun and Cook (2012) as present in Australia have not been supported by DNA sequence data. Molecular studies of Australian powdery mildews (e.g., Cunnington et al., 2003, 2004a,b, 2005a,b; Liberato and Cunnington, 2006, 2007; McTaggart et al., 2012) identified common exotic species on crops and wild plants that were introduced to Australia during European colonization, and had major impacts on the entire vegetation of the continent (Kirkpatrick, 1999; Cook and Dias, 2006; Randall, 2007; Fensham and Laffineur, 2019). A comprehensive molecular study of species of *Golovinomyces* collected from non-native asteraceous hosts concluded that all these powdery mildews were introduced to Australia from the northern hemisphere (Cunnington et al., 2010). Only three powdery mildew taxa have been considered native to Australia based on molecular studies, specifically, *Oidium ixodiae* on *Ixodia achillaeoides* (Cunnington et al., 2005a); *Neoerysiphe kerribeeensis*, on the indigenous *Senecio glossanthus*, and several other *Senecio* spp. introduced to Australia (Beilharz et al., 2010); and *Pseudoidium hardenbergiae* on *Hardenbergia* spp. (Cunnington et al., 2004b). However, Cunnington et al. (2004b) did not exclude the possibility that *Ps. hardenbergiae* was introduced to Australia from New Zealand, where it was originally described (Boesewinkel, 1977), while Braun and Cook (2012) noted that the asexual morphs on *Hardenbergia* spp. in Australia and North America may differ from *Ps. hardenbergiae*. Neither *O. ixodiae* nor *N. kerribeeensis* have been reported outside Australia. Curiously, another powdery mildew species known only from Australia is *Golovinomyces lycopersici* on tomato, a vegetable introduced from overseas (Kiss et al., 2001, 2005; Braun et al., 2019). This tomato pathogen is closely related to the Australian *O. ixodiae* (Cunnington et al., 2005a), and also to *G. longipes* that causes epidemics on solanaceous vegetables and ornamentals in Europe and the USA (Kiss et al., 2008; Kovács et al., 2011).

The distinction between native vs. introduced powdery mildews has special relevance for Australia, an island continent where the native vegetation has largely evolved in isolation until the start of European settlement in 1788 (Kirkpatrick, 1999; Randall, 2007; Fensham and Laffineur, 2019). That particular year is considered as a sharp biogeographic landmark in the history of the Australian flora of vascular plants (Fensham and Laffineur, 2019) as it marked the commencement of deliberate and accidental human-assisted introductions of altogether over 28,000 plant species from overseas, including agricultural and horticultural crops, ornamentals, and pasture species (Randall, 2007). Walker (1983) noted that until the early 1980s, most reports of powdery mildews in Australia were on dicots and monocots introduced from overseas after 1788, and only a few on natives. Walker (1983) also observed that all of the reports of powdery mildew infections on native plants in Australia were observed in artificial conditions, particularly glasshouses or nurseries. Clearly, the distribution and host range of powdery mildews in Australian vegetation needs to be considered in the light of the massive introduction of exotic plant species since 1788.

This study provides the first comprehensive database of powdery mildew species identified in Australia. Our objectives were to (i) compile an up-to-date list of all the powdery mildew taxa identified in Australia based on published DNA barcode sequences; and (ii) identify as many powdery mildew species as possible from across the country based on morphological characteristics and nrDNA ITS sequences. Special attention was paid to the identification of powdery mildews infecting native Australian plants to better understand the host ranges and global distribution of the Erysiphales.

## Materials and Methods

### Compiling a List of Powdery Mildews Identified in Australia Based on DNA Barcode Sequences Published Prior This Study

Searches were conducted in the NCBI nucleotide database, GenBank, with each genus name of the Erysiphales listed by Braun and Cook (2012), supplemented with “AND Australia” (e.g., “*Oidium* AND Australia”), to find all the publicly available DNA sequences for powdery mildew specimens collected in Australia. All the old and the currently accepted names of anamorphic and teleomorphic genera were included in these searches. Due to a number of reasons, some ITS sequences deposited in GenBank as fungal entries are inaccurate (Nilsson et al., 2006; Bidartondo, 2008; Irinyi et al., 2015; Hibbett et al., 2016; Selosse et al., 2016). Therefore, BLASTn searches were carried out with the retrieved entries to ensure that these represented powdery mildews.

When necessary, powdery mildew taxon names retrieved from GenBank were updated to correspond to the currently accepted nomenclature, e.g., for taxa belonging to the well-known species complex *G. orontii* (Braun et al., 2019). Records representing other well-known species complexes that have not been resolved from a taxonomic perspective, e.g. *B. graminis* (Braun and Cook, 2012), were considered as a single taxon. Those powdery mildew records in GenBank that have only been determined at the generic level were considered as representative of different species if their ITS sequences differed in more than 3 nucleotide positions based on the results obtained by Kovács et al. (2011).

### Field Collection, DNA Extraction, and Microscopic Examination of Specimens

Starting from 2017, powdery mildew-infected plant samples were collected from across Australia. Systematic surveys were conducted by four plant pathologists in Queensland and New South Wales, and *ad-hoc* surveys by 20 members of the National Plant Biosecurity Diagnostic Network. Powdery mildew mycelia were removed from the host plant surfaces with 1–1.5 cm^2^ pieces of cellotape, and genomic DNA was extracted using the buffers from an Extract-N-Amp Plant PCR kit (Sigma-Aldrich, St. Louis, MO) according to the manufacturer's instructions. A part of the powdery mildew material was examined and photographed under a light microscope whilst still fresh. All plant samples were dried and pressed as herbarium specimens, and then rehydrated in the laboratory for comprehensive morphological examinations if required. Their rehydration was conducted by boiling small pieces of infected plant materials in lactic acid (100% v/v) on a microscope slide, as described by Shin and La (1993). During microscopy with bright field, phase contrast, and differential interference contrast (DIC) optics, the following information was noted: shape and size of conidia, based on measurements of 25 conidia per specimen, presence or absence of fibrosin bodies in fresh conidia, nature of conidiogenesis, characteristics of the conidiophore, e.g., size and shape of foot cell, position of the basal septum, shape and position of hyphal appressoria, position of germ tubes of conidia, when found, and shape of appressoria on germ tubes of conidia. Droplets in conidia that appeared to be made of lipids were stained with 0.3% v/v alcoholic Sudan Black B solution by slightly warming the microscope slides over a flame before microscopic examination. Conidial germination in fresh powdery mildew samples collected from *Acalypha* spp. was studied on cellophane placed on 1.5% water agar (Szentiványi and Kiss, 2003), and the epidermis of onion scale (To-anun et al., 2005), after incubation at 25°C for 24, 48, 72, and 96 h. Representative specimens of each host-pathogen combination were deposited at the Queensland Plant Pathology Herbarium (BRIP), the New South Wales Plant Pathology and Mycology Herbarium (DAR), and the Victorian Plant Pathology Herbarium (VPRI).

### PCR Amplification of the ITS Region From Fresh Specimens

Amplification of the ITS region followed the nested PCR method described by Cunnington et al. (2003), with some modifications, and using the Extract-N-Amp Plant PCR kit. The first PCRs were carried out in 20 μL final volumes, consisting of 10 μL hot start PCR ReadyMix from the Extract-N-Amp Plant PCR kit, 1 μL of each powdery mildew-specific primers PMITS1 and PMITS2 (Table 1) at 10 μM, 6 μL ultrapure water, and 2 μL total genomic DNA. The nested reactions were also carried out in 20 μL final volumes, and consisted of 10 μL ReadyMix, 1 μL of each universal fungal primers ITS1-F (Gardes and Bruns, 1993) and ITS4 (White et al., 1990) at 10 μM, 7 μL ultrapure water, and 1 μL of the product of the first PCRs. The conditions for the first PCRs were as follows: 94°C for 10 min; 35 cycles of 1 min at 94°C, 1 min at 60°C, and 2 min at 72°C; and finally 10 min at 72°C. The nested PCRs consisted of 94°C for 5 min; 35 cycles of 45 s at 94°C, 45 s at 55°C, and 1 min at 72°C; and finally 10 min at 72°C. PCR products of the nested reactions were purified and sequenced by Macrogen Inc. (Seoul, Korea) with primers ITS1-F and ITS4.

**Table 1:** The powdery mildew-specific, and other specific rDNA primers used in this work.

**Primer designation**	**Orientation**	**Sequence (5^′^ → 3^′^)**	**Purpose**	**References**
PMITS1	Forward	TCG GAC TGG CCY AGG GAG A	Amplification of the ITS region and a part of the flanking 18S and 28S rDNA regions in most powdery mildews	Cunnington et al., 2003
PMITS2	Reverse	TCA CTC GCC GTT ACT GAG GT		Cunnington et al., 2003
PM-Ei-F1	Forward	CCG TGT CGA TTT GTA TCG TG	Amplification of a large part of the entire ITS region in *Erysiphe izuensis*	This paper
PM-Ei-R1	Reverse	ACT CTG TCG CGA GAA GCA AG		This paper
T3	Reverse	ACG CTC GAA CAG GCA TGC CC	Binding site at the 3′-end of the 5.8S rDNA region of most powdery mildews and some other fungi	Hirata and Takamatsu, 1996
PM5	Forward	TTG CTT TGG CGG GCC GGG	Binding site in the ITS1 region of most powdery mildews	Takamatsu and Kano, 2001
TW14	Reverse	GCT ATC CTG AGG GAA ACT TC	Binding site at the 3′-end of the D1-D2 domain of the 28S rDNA region in most powdery mildews and some other fungi	https://nature.berkeley.edu/brunslab/tour/primers.html#28s
T4	Forward	TCA ACA ACG GAT CTC TTG GC	Binding site at the 5′-end of the 5.8S rDNA region in most powdery mildews and some other fungi	Hirata and Takamatsu, 1996
T2	Forward	GGG CAT GCC TGT TCG AGC GT	Binding site at the 3′-end of the 5.8S rDNA region in most powdery mildews and some other fungi	Hirata and Takamatsu, 1996
Pm-LSU-R2	Reverse	ACT CCA AGG GAG CCA GAT TT	Amplification of the D1-D2 domain of the 28S rDNA in *Salmonomyces*, in combination with the universal primer LR3	This paper
Pm-LSU-R3	Reverse	GCT TTA CAT AGG CGC AGG TC		This paper

### PCR Amplification of an ITS Fragment of *E. izuensis* With Specific Primers Designed in This Study

Amplifications from fresh powdery mildew colonies sampled from the leaves of *Rhododendron* spp. as described above, consistently yielded ITS sequences that were 98–99% similar to those of a number of uncultured fungi reported from environmental samples. As the powdery mildew species infecting the examined *Rhododendron* leaves was identified as *E. izuensis* based on the morphology of the asexual and sexual morphs, species-specific primers PM-Ei-F1 and PM-Ei-R1 (Table 1) were designed based on the ITS sequence of this species available in GenBank under accession number LC009975 using the software Primer3 (Untergasser et al., 2012). Instead of ITS1-F/ITS4, the *E. izuensis*-specific PM-Ei-F1/PM-Ei-R1 primer pair was used to amplify an ~450 bp long fragment, including the partial ITS1, complete 5.8S, and partial ITS2 regions in the powdery mildew samples from *Rhododendron* spp. using the nested PCR protocol described above. PCR products were sequenced by Macrogen Inc. with primers PM-Ei-F1 and PM-Ei-R1.

### DNA Extraction and PCR Amplification of the ITS Region From Herbarium Specimens Collected Between 1975 and 2013 in Australia

During a review of older powdery mildew specimens available at BRIP, collected between 1975 and 2013, 30 specimens were selected based on the amount and quality of the powdery mildew mycelium available on plant tissues, assessed under a dissecting microscope. Approximately 20 mg of infected plant tissue was taken from dried herbarium specimens, and disrupted in a Tissue Lyser (QIAGEN) with 2.5 mm glass beads (Daintree Scientific). Genomic DNA was extracted with the DNeasy Plant Mini Kit (QIAGEN) according to the manufacturer's instructions, with the modification that N-phenacylthiazolium bromide (PTB; Epichem, Perth) was added to the lysis buffer (final concentration of 2.5 mM). PTB is known to enhance DNA purification efficiency from herbarium specimens (Lister et al., 2008; Telle and Thines, 2008). Amplification of the ITS region was as described above for fresh specimens, with the exception that the PCR was performed with Phusion® High-Fidelity PCR Master Mix with HF Buffer (New England Biolabs), supplemented with Bovine Serum Album (New England Biolabs; final concentration of 1 μg/μL). Purification of the nested PCR products and sequencing was as described above for fresh specimens.

### Study of Herbarium Specimens From China and Argentina

Powdery mildew specimens collected from *Acalypha* spp. in China and Argentina (Supplementary Table 1) were rehydrated during boiling in lactic acid and observed under a light microscope as described above. PCR amplifications of the ITS region were carried out according to the protocol described above.

### Sequencing the ITS Region in Powdery Mildew Specimens Collected From *Acalypha* spp. Following Cloning the PCR Product

Chromatograms obtained from direct sequencing of the ITS region in all the fresh powdery mildew specimens collected from *Acalypha* spp. in Australia contained double peaks at certain positions. Since intra-sample variation in ITS sequences of powdery mildew species has been reported previously (Kovács et al., 2011), the ITS region of two specimens, one from a young powdery mildew colony infecting *A. nemorum*, and the other from a young colony from *A. wilkesiana*, were sequenced after cloning, to reveal potential intra-sample ITS variants. The nested PCR products from these two specimens were cloned at Macrogen Inc. using TA cloning. Ten recombinant plasmids per PCR product were sequenced with the primer M13F.

### PCR Amplification of the 28S and 18S nrDNA Regions in Powdery Mildew Specimens Collected From *Acalypha* spp.

The 18S nrDNA region was amplified in two steps: the first PCR was carried out with the universal fungal primer NS1 (White et al., 1990) and the powdery mildew-specific primer T3 (Hirata and Takamatsu, 1996), followed by two sets of semi-nested PCRs, one with the universal fungal primers NS1 and ITS2, and another one with NS1 and NS8 (White et al., 1990). The 5′-end of the 28S rDNA (including domains D1 and D2) was also amplified in two steps, the first being PCRs with primers PM5 and TW14, followed by two sets of semi-nested PCRs, one with primers T4 and TW14, and another one with T2 (Hirata and Takamatsu, 1996) and TW14. All the specific primers used in this work are listed in Table 1.

The composition of the PCR mixes was as described above for the ITS PCRs from fresh powdery mildew specimens. The parameters for the first PCRs were as follows: 94°C for 10 min; 35 cycles of 1 min at 94°C, 1 min at 52°C, and 2 min at 72°C; and finally 10 min at 72°C. The semi-nested PCRs consisted of 94°C for 5 min; 35 cycles of 1 min at 94°C, 1 min at 52°C, and 1 min at 72°C; and finally 10 min at 72°C. All the products of the semi-nested PCRs were purified and sequenced at Macrogen Inc., using primers NS1, NS8, and ITS2 for the 18S products, and primers T2, T4, and TW14 for the 28S products.

A poly-A/T motif in the ITS2 region made all the chromatograms of the 28S products unreliable starting from that motif; therefore, two new nested reverse primers, Pm-LSU-R2 and Pm-LSU-R3 (Table 1) were designed at ~180 and 300 bp downstream of the repeat region, using Primer3, to enable re-sequencing of the repetitive regions to achieve reliable consensus sequences. Each of these new reverse primers was used in combination with the universal fungal primer LR3 (Vilgalys and Hester, 1990) in nested PCRs, using the PCR products from the first 28S PCRs as templates. The products of these nested PCRs were purified and sequenced at Macrogen Inc. with primers LR3, Pm-LSU-R2, and Pm-LSU-R3.

### Initial Sequence Analyses

Sequences were compiled from chromatograms, following visual inspections for potential polymorphisms. In order to minimize false positives arising from potential sequencing errors, stringent quality control was conducted. To obtain a reliable consensus sequence, single nucleotide polymorphisms were only accepted if the base call quality was at least 30, and also the polymorphism occurred in more than one sequence (James et al., 2009). Consensus sequences were produced by trimming and assembling the forward and reverse sequences using Geneious Prime 2019.1.3 (Biomatters Ltd.).

### Phylogenetic Analyses of 18S, 28S, and 5.8S Sequences to Investigate the Generic Placement of the Powdery Mildews Infecting *Acalypha* spp. Within the Erysiphales

To reveal the generic placement of the powdery mildews collected from *Acalypha* spp. within the Erysiphales, a three-locus phylogenetic analysis was conducted using sequences of the nrDNA 28S, 18S, and 5.8S regions. Sequences of representative specimens of all but one of the 18 powdery mildew genera accepted to date were retrieved from GenBank (Supplementary Table 2). The genus *Takamatsuella*, for which no authentic sequence data is available, could not be included in this analysis. In *Brasiliomyces*, there are only two specimens of *Br. malachrae* that have been included in molecular studies so far; a large part of 18S, together with the ITS region was sequenced from specimen MUMH 3119, while the ITS and a large part of 28S was determined from specimen MUMH 3093 (Cabrera et al., 2018). The sequence available for the latter specimen was included in the analysis, and the nucleotides for the 18S sequence were coded as missing. This was also done in the case of all the four powdery mildew specimens known from *Acalypha* spp. to date, and described as *Ps. javanicum* (Meeboon and Takamatsu, 2015), as the 18S sequences are not available for these specimens (Supplementary Table 2).

All sequence alignments were generated using MAFFT v. 7.388 (Katoh and Standley, 2013), and visually inspected for potential misalignments or ambiguously-aligned regions. Due to the variable size of the 28S and 18S sequences retrieved from GenBank, alignments for these loci were trimmed to the length of the shortest sequence. For the multi-locus phylogenetic analysis, congruency of loci was tested through incongruence-length difference (ILD) partition homogeneity test (Farris et al., 1995) implemented in PAUP v. 4.0b10 (Swofford, 2002), which detected no significant conflict in tree topologies (*P*-value = 1.0), and allowed concatenation of loci.

Phylogenetic analyses were conducted using two methods; Bayesian Inference (BI) and Maximum likelihood (ML). For BI, the best-fit nucleotide substitution model for each locus was determined by comparing the Akaike Information Criterion using PAUP v. 4.0 (Swofford, 2002) and MrModeltest v. 2.3 (Nylander, 2004). Two Markov Chain Monte Carlo (MCMC) chains were run using MrBayes v. 3.2.4 (Ronquist and Huelsenbeck, 2003). One tree per 100 generations was saved, and the runs were ended when the standard deviation of split frequencies reached below 0.01. The 50% majority rule consensus tree was estimated after a 25% burn-in of the saved trees. Maximum likelihood analysis was conducted in RAxML v. 8.2.11 (Stamatakis, 2014) using the GTRGAMMA model applied to the individual partitions, with 1,000 bootstrap replicates. Alignments and trees were deposited in TreeBASE (submission no. 25949).

### Phylogenetic Analyses of ITS Sequences of Specimens Belonging to Different Genera

ITS sequences of powdery mildew species belonging to distantly related genera are too divergent to allow for non-ambiguous alignment of all sequences (Takamatsu et al., 1998, 2015a; Mori et al., 2000). Therefore, the ITS sequences determined in this study were grouped in the following four subsets of closely related taxa: (i) the *Microsphaera* lineage of the genus *Erysiphe*; (ii) *Podosphaera* and *Sawadaea* species; (iii) *Golovinomyces, Neoerysiphe, Arthrocladiella* and *Microidium* species; and (iv) *Leveillula* species. At least 95% similar ITS sequences coming from similar taxa collected overseas were added to each of these four subsets based on BLAST searches (Supplementary Table 3), and each subset of sequences was analyzed separately. No ITS analysis was performed for *Blumeria* as this genus was represented by only three specimens collected in Australia, and *Blumeria* is only distantly related to all the other genera of the Erysiphales. Similarly, sequences of *E. necator* and *E. australiana* were not included in our phylogenetic analyses because these taxa belong to the *Uncinula* lineage of the genus *Erysiphe* as defined by Takamatsu et al. (2015b), and their ITS sequences are too divergent to be analyzed together with those of the *Microsphaera* lineage of *Erysiphe* (Takamatsu et al., 2015a).

Alignments of the four subsets of ITS sequences were generated using MAFFT v. 7.388 (Katoh and Standley, 2013), and visually inspected for potential misalignments or ambiguously-aligned regions. Phylogenetic analyses were conducted using BI and ML methods as described above. Alignments and trees were deposited in TreeBASE (submission no. 25949).

## Results

### Powdery Mildews Identified in Australia Based on Their ITS Sequences Published Prior This Study

As of February 2020, the identity of 32 powdery mildew taxa collected from over 60 host plant species in Australia was supported by partial or complete ITS sequences deposited in GenBank (Table 2). DNA barcodes other than the ITS sequences were not available in GenBank for any of these specimens. Those 32 taxa belonged to eight genera, and included 30 well-defined species, and two more taxa identified only to genus level, namely, *Pseudoidium* sp. on *Convolvulus erubescens*, closely related to *E. heraclei* and *E. convolvuli* based on ITS analyses (Cunnington et al., 2003), and *Euoidium* sp. on *Nicotiana alata* and *Solanum melongena*, with ITS sequences identical to those of *G. lycopersici* and *G. longipes* (Cunnington et al., 2005b). Therefore, we considered that 30 powdery mildew species were reliably identified based on their ITS sequences in Australia prior to our study.

**Table 2:** List of powdery mildews identified in Australia based on nrDNA ITS sequences and morphological characteristics.

**Powdery mildew genera/species**	**Host plant**	**Herbarium accession number**	**Place and date of collection**	**ITS GenBank accession number**	**References**
*ARTHROCLADIELLA*					
*A. mougeotii*	*Lycium barbarum*	VPRI 18039	Bridgewater, VIC, July 1992	AF073358	Cunnington et al., 2003
	*Lycium barbarum*	BRIP 66057	Killarney, QLD, April 2017	MF496139	Kiss et al., 2018a
	*Lycium barbarum*	BRIP 68795	Killarney, QLD, October 2017	MT174179	This paper
*BLUMERIA*					
*Blumeria graminis*	*Hordeum vulgare*	VPRI 18279	Horsham, VIC, September 1992	AF073352	Cunnington et al., 2003
	*Hordeum vulgare*	BRIP 68826	Gatton, QLD, June 2017	MT174180	This paper
	***Phalaris canariensis***	**BRIP 52847**	**Condamine Plains, QLD, September 2009**	**MT174181**	**This paper**
*ERYSIPHE*					
*E. alphitoides*	***Jagera pseudorhus***	**BRIP 68798**	**Mt. Tamborine, QLD, December 2017**	**MT174182**	**This paper**
	***Jagera pseudorhus***	**BRIP 68799**	**Burleigh Heads, QLD, July 2018**	**MT174183**	**This paper**
	*Mangifera indica*	VPRI 20379	Tweed Valley, NSW, October 1994	AB237799	Limkaisang et al., 2006
	*Mangifera indica*	VPRI 20364	Hopkins Creek, QLD, October 1994	AB237798	Limkaisang et al., 2006
	*Mangifera indica*	VPRI 18420	Indooroopilly, QLD, October 1992	AB237795	Limkaisang et al., 2006
	*Quercus robur*	BRIP 68796	Toowoomba, QLD, October 2017	MT174184	This paper
	*Quercus* sp.	VPRI 18763	Mitcham, VIC, January 1993	AB292705	Takamatsu et al., 2007
	*Quercus* sp.	VPRI 20423	The Basin, VIC, December 1994	AB292704	Takamatsu et al., 2007
*E. aquilegiae*	*Aquilegia* sp.	VPRI 20820	Parkville, VIC, March 1996	AY452800	Cunnington et al., 2004a
	*Catharanthus roseus*	BRIP 46649, VPRI 32380	Brisbane, QLD, July 2005	DQ335569	Liberato and Cunnington, 2006
	*Delphinium* sp.	VPRI 19613	Panton Hill, VIC, November 1993	AY452802	Cunnington et al., 2004a
	*Nigella damascena*	VPRI 18533	Burnley, VIC, December 1992	AY452804	Cunnington et al., 2004a
	*Ranunculus* sp.	VPRI 18740	Pheasant Creek, VIC, January 1993	AY452805	Cunnington et al., 2004a
*E. australiana*	*Lagerstroemia indica*	VPRI 21732	Hawthorn East, VIC, February 1998	AF073347	Cunnington et al., 2003
	*Lagerstroemia indica*	BRIP 68797	Geebung, QLD, March 2017	MT174185	This paper
	*Lagerstroemia indica*	BRIP 48722	Sherwood, December 2006	MT174186	This paper
***E. cruciferarum***	***Brassica juncea***	**BRIP 69033**	**Jondaryan, QLD, November 2017**	**MT174187**	**This paper**
*E. diffusa*	***Glycine clandestina***	**BRIP 68827**	**Rangeville, QLD, June 2018**	**MT174188**	**This paper**
	*Glycine max*	BRIP 55388	QLD, 2012	JX136797	McTaggart et al., 2012
	*Glycine max*	BRIP 58458	Nangwee, QLD, 2013	MT174189	This paper
	*Glycine max*	BRIP 62030	Giru, QLD, 2014	MT174190	This paper
	*Glycine max* cv. Jackson	BRIP 68997	Grafton, NSW, April 2018	MT174191	This paper
***E. euonymicola***	***Euonymus japonicus***	**BRIP 69034**	**Toowoomba, QLD, November 2017**	**MT174192**	**This paper**
*E. heraclei*	***Anethum graveolens***	**BRIP 59438**	**Mareeba, QLD, July 2013**	**MT174193**	**This paper**
	***Anethum graveolens***	**BRIP 68828**	**Toowoomba, QLD, December 2017**	**MT174194**	**This paper**
	*Daucus carota*	BRIP 68829	Toowoomba, QLD, December 2017	MT174195	This paper
	*Daucus carota*	VPRI 41227	Murrumbidgee, NSW, August 2007	EU371725	Cunnington et al., 2008b
***E. izuensis***	***Rhododendron*** **sp**.	**BRIP 69724**	**Toowoomba, QLD, June 2018**	**MT174196**	**This paper**
	***Rhododendron indicum***	**BRIP 68833**	**Toowoomba, QLD, August 2018**	**MT174197**	**This paper**
	***Rhododendron*** **sp**.	**BRIP 70493**	**Mt. Coot-tha, QLD, July 2018**	**MT174198**	**This paper**
*E. necator*	*Vitis vinifera*	VPRI 19719	Dandenong, VIC, December 1993	AF073346	Cunnington et al., 2003
	*Vitis vinifera* cv. Fiano	BRIP 69725	Stanthorpe, QLD, December 2018	MT174232	This paper
*E. pisi*	*Pisum sativum*	VPRI 19688	Hopetoun, VIC, November 1993	AF073348	Cunnington et al., 2003
*E. platani*	*Platanus x hybrida*	BRIP 68800	Adelaide, SA, March 2018	MT174199	This paper
	*Platanus x hybrida*	DAR 83490	Adelaide, SA, 2018	MT174200	This paper
	*Platanus occidentalis*	VPRI 21733	Melbourne, VIC, February 1998	AF073349	Cunnington et al., 2003
*E. quercicola*	*Acacia holosericea*	VPRI 20468	Humpty Doo, NT, February 1995	AB237806	Limkaisang et al., 2006
	*Acacia mangium*	VPRI 20374	Tully, QLD, October 1994	AB237807	Limkaisang et al., 2006
	*Acacia mangium*	VPRI 20907	Mission Beach, QLD, June 1993	AB237808	Limkaisang et al., 2006
	*Eucalyptus camaldulensis*	VPRI 19251	West Pennant Hills, NSW, May 1993	AB237796	Limkaisang et al., 2006
	*Mangifera indica*	VPRI 20332	Douglas, QLD, August 1994	AB237797	Limkaisang et al., 2006
	*Quercus robur*	VPRI 19013	Stirling, SA, March 1993	AB295454	Takamatsu et al., 2007
	*Quercus* sp.	VPRI 20422	The Basin, VIC, December 1994	AB295455	Takamatsu et al., 2007
*E. syringae*	*Syringa vulgaris*	VPRI 41368	Ormond, VIC, March 2008	FJ755790	Cunnington and Brett, 2009
	*Syringa vulgaris*	VPRI 43062	VIC, February 2018	MH368484	GenBank
***E***. **cf**. ***trifoliorum***	***Acacia orites***	**BRIP 70580**	**Canungra, QLD, April 2019**	**MT174201**	**This paper**
	***Pisum sativum***	**BRIP 68831**	**Killarney, QLD, November 2017**	**MT174202**	**This paper**
	***Vicia tetrasperma***	**BRIP 68838**	**Tipton, QLD, Oct 2017**	**MT174203**	**This paper**
*Erysiphe* sp.	*Araujia sericifera*	BRIP 66128	Rosebery, NSW, July 2017	MG551720	Southwell et al., 2018
	*Araujia sericifera*	BRIP 66129	Chiefley, NSW, July 2017	MG551721	Southwell et al., 2018
	*Araujia sericifera*	BRIP 68832	Rangeville, QLD, March 2018	MT174204	This paper
	***Cassia fistula***	**BRIP 68834**	**Toowoomba, QLD, June 2018**	**MT174205**	**This paper**
*Pseudoidium hardenbergiae*	*Hardenbergia* sp.	VPRI 19879	Devenport, TAS, March 1994	AY450959	Cunnington et al., 2004b
***Pseudoidium hortensiae***	***Hydrangea macrophylla***	**BRIP 68852**	**Toowoomba, QLD, January 2018**	**MT174206**	**This paper**
*Pseudoidium* sp.	*Convolvulus erubescens*	VPRI 20708	Flinders ranges, SA, July 1987	AF154328	Cunnington et al., 2003
*GOLOVINOMYCES*					
*Euoidium* sp.	*Nicotiana alata*	VPRI 19196	Burnley, VIC, May 1993	AY683041	Cunnington et al., 2005b
	*Solanum melongena*	VPRI 17907	Adelaide, SA, May 1992	AY683038	Cunnington et al., 2005b
***G. ambrosiae***	*Persicaria decipiens*	HAL 3274 F, BRIP 69035	Redwood Park, QLD, November 2017	MH745099	Braun et al., 2019
*G. biocellatus*	*Mentha* sp.	BRIP 46650, VPRI 32381	Brisbane, QLD, July 2005	EU035602	Liberato and Cunnington, 2007
	*Salvia* sp.	VPRI 18671	Stanthorpe, QLD, January 1993	AF154323	Cunnington et al., 2003
	*Solanum lycopersicum*	VPRI 19373	St. Lucia, QLD, July 1993	AY683040	Cunnington et al., 2005b
***G. bolayi***	***Capsella bursa-pastoris***	**BRIP 68842**	**Toowoomba, QLD, June 2018**	**MT174213**	**This paper**
	***Solanum tuberosum***	**BRIP 50498**	**Gatton, QLD, September 2007**	**MT174214**	**This paper**
*G. cichoracearum*	*Aster subulatus*	VPRI 17692	Ardmona, VIC, October 1991	GQ183937	Cunnington et al., 2010
	*Leucanthemum paludosum (*syn. *Chrysanthemum paludosum)*	VPRI 20465	Brisbane, QLD, January 1995	GQ183947	Cunnington et al., 2010
	*Dahlia* sp.	VPRI 21385	Burwood, VIC, April 1997	GQ183949	Cunnington et al., 2010
	*Solidago* sp.	VPRI 19021	Burnley, VIC, March 1993	GQ183940	Cunnington et al., 2010
	*Tanacetum balsamita*	VPRI 19067	Olinda, VIC, March 1993	GQ183942	Cunnington et al., 2010
	*Tanacetum parthenium*	VPRI 18942	Dromana, VIC, March 1993	GQ183939	Cunnington et al., 2010
	*Tanacetum parthenium*	BRIP 68841	Sydney, NSW, September 2017	MT174215	This paper
	*Tanacetum vulgare*	VPRI 20223	Mt. Coot-tha, QLD, August 1994	GQ183945	Cunnington et al., 2010
	*Zinnia* sp.	VPRI 19824	Toowoomba, VIC, February 1994	GQ183944	Cunnington et al., 2010
*G. glandulariae*	*Glandularia aristigera*	BRIP 70490	Bunya Mountains, QLD, July 2019	MN190239	Crous et al., 2019
	*Glandularia aristigera*	BRIP 70491	Bunya Mountains - Maclagan Road, QLD, June 2019	MN190241	Crous et al., 2019
	*Glandularia aristigera*	BRIP 70492	Bunya Mountains, QLD, February 2017	MN190240	Crous et al., 2019
	*Glandularia aristigera*	BRIP 68801	Bunya Mountains, QLD, March 2018	MN190242	Crous et al., 2019
	*Glandularia aristigera*	BRIP 70531	Bunya Mountains, QLD, June 2019	MN190243	Crous et al., 2019
*G. ixodiae*	*Ixodia achillaeoides*	VPRI 20703	Lenswood, SA, September 1995	AY769954	Cunnington et al., 2005a
***G. latisporus***	***Aster subulatus***	**BRIP 68839**	**Jondaryan, QLD, November 2017**	**MT174207**	**This paper**
	***Helianthus annuus***	**BRIP 68840**	**Toowoomba, QLD, April 2018**	**MT174208**	**This paper**
	***Helianthus annuus***	**BRIP 28996**	**Toowoomba, QLD**,	**MT174209**	**This paper**
	***Helianthus annuus***	**BRIP 49653**	**Gatton, QLD, May 2007**	**MT174210**	**This paper**
	***Helianthus*** **sp**.	**BRIP 66781**	**Kununurra, WA, July 2017**	**MT174211**	**This paper**
	***Xanthium strumarium***	**BRIP 11128**	**Nebo, QLD, July 1975**	**MT174212**	**This paper**
*G. lycopersici*	*Solanum lycopersicum*	VPRI 19847	Timmering, VIC, February 1994	AF229021	Kiss et al., 2001
	*Solanum lycopersicum*	DAR 70008	Lauderdale, TAS, 1994	HQ286673	Kovács et al., 2011
	*Solanum lycopersicum*	DAR 35763	White Hills, Bendigo, VIC, 1980	EU327330	Kiss et al., 2008
	*Solanum lycopersicum*	DAR 71625	Unknown locality, SA, 1996	EU327332	Kiss et al., 2008
	*Solanum lycopersicum*	BRIP 68830	Maclean, NSW, December 2017	MT174216	This paper
*G. orontii*	*Penstemon serrulatus*	VPRI 19066	Olinda, VIC, March 1993	GQ183941	Cunnington et al., 2010
	*Phyla nodiflora*	VPRI 20467	Brisbane, QLD, February 1995	GQ183948	Cunnington et al., 2010
	*Solanum tuberosum*	VPRI 20740	Toolangi, VIC, October 1995	AY683039	Cunnington et al., 2005b
	*Taraxacum officinale*	VPRI 19707	Kingswood, SA, November 1993	GQ183943	Cunnington et al., 2010
	*Viola* sp.	VPRI 18422	Parkville, VIC, November 1992	GQ183938	Cunnington et al., 2010
*LEVEILLULA*					
*L. taurica*	*Capsicum annuum*	VPRI 20146	Adelaide, SA, June 1994	AF073351	Cunnington et al., 2003
	*Capsicum annuum*	BRIP 63342	Gumlu, QLD, September 2015	MT174217	This paper
	***Euphorbia cyathophora***	**BRIP 68802**	**Iluka, NSW, December 2017**	**MT174218**	**This paper**
	***Euphorbia heterophylla***	**BRIP 66778**	**Iama, Torres Strait, May 2017**	**MT174219**	**This paper**
	***Solanum lycopersicum***	**BRIP 68843**	**Carnarvon, WA, May 2018**	**MT174220**	**This paper**
***MICROIDIUM***					
***M. phyllanthi***	***Phyllanthus debilis***	**BRIP 68803**	**Cairns, QLD, July 2018**	**MT174221**	**This paper**
*NEOERYSIPHE*					
*N. galeopsidis*	*Ajuga reptans*	VPRI 19007	Melbourne, VIC, 23 March 1993	AF073357	Cunnington et al., 2003
*N. kerribeeensis*	*Senecio glossanthus*	DAR 33493	Kerribee Station, NSW, September 1978	GU356546	Beilharz et al., 2010
	*Senecio glossanthus*	VPRI 21786	Natimuk, VIC, October 1995	GU356545	Beilharz et al., 2010
	*Senecio minimus (syn. Erechtites minimus)*	VPRI 19119	Kinglake N.P., VIC, April 1993	GU356541	Beilharz et al., 2010
	*Senecio* sp.	VPRI 19944	Hobart, TAS, March 1994	GU356544	Beilharz et al., 2010
	*Senecio* sp.	VPRI 19929	Devonport, TAS, March 1994	GU356543	Beilharz et al., 2010
	*Senecio* sp.	VPRI 19834	Olinda, VIC, February 1994	GU356542	Beilharz et al., 2010
	*Senecio vulgaris*	VPRI 18591	Burnley, VIC, December 1992	GU356540	Beilharz et al., 2010
*PODOSPHAERA*					
*P. aphanis*	*Fragaria x ananassa*	VPRI 19031	Toolangi, VIC, March 1993	AF073355	Cunnington et al., 2003
*P. fusca*	*Calendula officinalis*	VPRI 20625	North Carlton, VIC, July 1995	AF154324	Cunnington et al., 2003
*P. leucotricha*	*Malus domestica*	VPRI 17729	Lake Eucumbene, NSW, Nov 1991	AF073353	Cunnington et al., 2003
	*Malus pumila*	VPRI 18381	Applethorpe, QLD, October 1992	e.g., MT178379	Smith et al., 2020
	*Malus domestica*	VPRI 18536	Taroona, TAS, November 1992	e.g., MT178355	Smith et al., 2020
	*Malus domestica*	VPRI 18575	Beaconsfield, TAS, December 1992	e.g., MT178381	Smith et al., 2020
	*Malus sylvestris*	VPRI 19785	Kingswood, SA, January 1994	e.g., MT178378	Smith et al., 2020
	*Malus* sp.	VPRI 19947	Hobart, TAS, March 1994	e.g., MT178367	Smith et al., 2020
***P. pannosa***	***Rosa*** **sp**.	**BRIP 68844**	**Highfields, QLD, June 2017**	**MT174222**	**This paper**
***P. plantaginis***	***Plantago lanceolata***	**BRIP 68845**	**Toowoomba, QLD, May 2018**	**MT174223**	**This paper**
	***Lactuca serriola***	**BRIP 68851**	**Carnarvon, WA, May 2018**	**MT174224**	**This paper**
*P. tridactyla*	*Prunus armeniaca*	VPRI 19864	Knoxfield, VIC, March 1994	AY833657	Cunnington et al., 2005c
	*Prunus cerasifera*	VPRI 19238	Taroona, TAS, May 1993	AY833656	Cunnington et al., 2005c
	*Prunus persica*	VPRI 19866	Knoxfield, VIC, March 1994	AY833651	Cunnington et al., 2005c
	*Prunus persica*	VPRI 19591	Burnley, VIC, November 1982	AY833653	Cunnington et al., 2005c
	*Prunus* sp.	VPRI 19006	Burnley, VIC, March 1993	AF154321	Cunnington et al., 2003
*P. xanthii*	*Cephalotus follicularis*	VPRI 41238	Keysborough, VIC, September 2007	EU367960	Cunnington et al., 2008a
	*Citrullus lanatus*	DAR 83497	Mascot, NSW, 2017	MT174225	This paper
	*Cucurbita maxima*	BRIP 69723	Unknown locality, NSW, 2017	MT174226	This paper
	*Dahlia pinnata*	BRIP 68846	Preston, QLD, May 2018	MT174227	This paper
	*Euryops chrysanthemoides*	BRIP 46314	Unknown locality, QLD, 2005	DQ205330	Liberato et al., 2006
	*Glandularia aristigera*	BRIP 70490	Bunya Mountains, QLD, July 2019	MN190026	Crous et al., 2019
	*Glandularia aristigera*	BRIP 70491	Bunya Mountains - Maclagan Road, QLD, June 2019	MN190028	Crous et al., 2019
	*Glandularia aristigera*	BRIP 70492	Bunya Mountains, QLD, February 2017	MN190027	Crous et al., 2019
	*Glandularia aristigera*	BRIP 68801	Bunya Mountains, QLD, March 2018	MN190029	Crous et al., 2019
	*Glandularia aristigera*	BRIP 70531	Bunya Mountains, QLD, June 2019	MN190244	Crous et al., 2019
	***Trema tomentosa***	**BRIP 70494**	**Canungra, QLD, 8 July 2018**	**MT174228**	**This paper**
	***Trema tomentosa***	**BRIP 70495**	**Canungra, QLD, 25 April 2019**	**MT174229**	**This paper**
	*Vigna radiata*	VPRI 19910	Formartin, QDL, March 1994	AY450961	Cunnington et al., 2004b
	*Vigna radiata*	BRIP 68847	Warwick, QLD, April 2017	MT174230	This paper
	*Vigna unguiculata*	VPRI 18815	Berrimah, NT, February 1993	AY450960	Cunnington et al., 2004b
***SALMONOMYCES***					
***S. acalyphae***	***Acalypha nemorum***	**BRIP 68805**	**Ravensbourne, QLD, June 2018**	**MT133545**	**This paper**
	***Acalypha wilkesiana***	**BRIP 68804**	**Brisbane, QLD, July 2018**	**MT133544**	**This paper**
*SAWADAEA*					
*S. bicornis*	*Acer negundo*	VPRI 19684	Duffy, ACT, November 1993	AF073356	Cunnington et al., 2003
***S. polyfida***	***Acer palmatum***	**BRIP 68806**	**Toowoomba, QLD, May 2018**	**MT174231**	**This paper**

*Powdery mildew species/genera, and/or their host plants that were recorded for the first time in Australia based on open access molecular data are shown in bold*.

### Powdery Mildews Identified in This Study

Our field surveys resulted in the collection of over 300 fresh powdery mildew specimens from 35 host plant species from across Australia. In addition, 30 herbarium specimens from BRIP, collected from 24 host plant species between 1975 and 2013, were also included in this study. All powdery mildew specimens were identified based on their morphology and ITS sequences. Altogether, these represented 25 species from nine genera (Table 2). Some of these powdery mildew species have already been reported from Australia based on their ITS sequences; another 12 species have not been identified with publicly available DNA sequences in Australia prior to our study.

In total, this study found 42 reliably identified powdery mildew species in 10 genera on over 90 host plant species across 68 plant genera in Australia (Table 2). Amongst these, a total of 26 plant species are reported here for the first time as hosts of several powdery mildews in Australia. Four of these newly reported host plants are native, i.e., *Acacia orites, Acalypha nemorum, Jagera pseudorhus*, and *Trema tomentosa*. Earlier studies have identified powdery mildews on other native hosts, i.e., *Acacia* spp., *Eucalyptus camaldulensis* (Limkaisang et al., 2006), *Cephalotus follicularis* (Cunnington et al., 2008a), *Convolvulus erubescens* (Cunnington et al., 2003), *Hardenbergia* sp. (Cunnington et al., 2004b), *Ixodia achilleoides* (Cunnington et al., 2005a), and *Senecio glossanthus* and *S. minimus* (Beilharz et al., 2010) (Table 2). A number of powdery mildews that are common on their host plants in many parts of the world, such as *E. cruciferarum* on oilseed brassicas (e.g., *B. juncea*), *E. heraclei* on dill (*Anethum graveolens*), *G. latisporus* on sunflower (*Helianthus annuus*), and *P. pannosa* on rose (*Rosa* sp.), are reported here for the first time based on molecular identifications, although these species could be identical to those recorded in Australia much earlier, under various names (Goss, 1964; Simmonds, 1966; Walker, 1983; Uloth et al., 2016).

**Figure 1:**
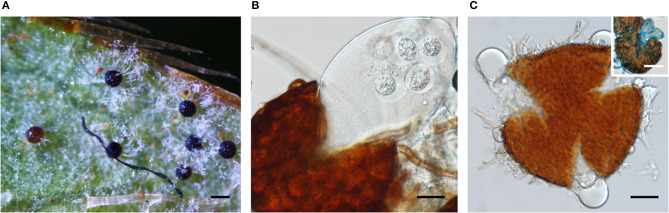
Chasmothecia of three powdery mildew species observed in this study. **(A)** *Erysiphe izuensis* on *Rhododendron* sp. (BRIP 69724). Bar = 100 μm. **(B)** *Podosphaera plantaginis* on *Plantago lanceolata* (BRIP 68845) Note the production of a single ascus in the chasmothecium. Bar = 25 μm. **(C)** *Golovinomyces latisporus* on *Helianthus annuus* (BRIP 28996) Bar = 20 μm. Inset: a chasmothecium with two asci stained with cotton blue. Bar = 50 μm.

The sexual morphs of three powdery mildew species, i.e., *E. izuensis* infecting *Rhododendron* sp., *G. latisporus* on *Helianthus annuus*, and *P. plantaginis* on *Plantago lanceolata* (Figure 1) were documented for the first time in Australia. Earlier, chasmothecia were reported for *E. necator* (Wicks and Magarey, 1985; Magarey et al., 1997), and *Neoerysiphe kerribeeensis* (Beilharz et al., 2010). Walker (1983) listed a few more powdery mildew species producing chasmothecia in Australia, including *B. graminis, E. australiana*, and *P. leucotricha*, without details of the host plant species, collection dates, and localities.

### Description of a Newly Recognized Lineage of the Erysiphales as a Reintroduced Genus

**Figure 2:**
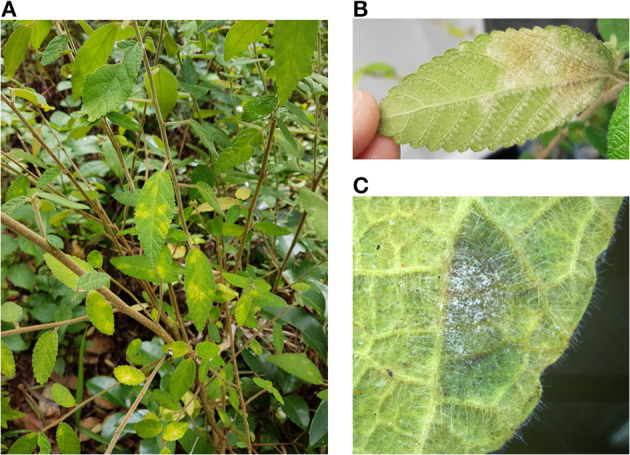
*Acalypha nemorum* infected with *Salmonomyces acalyphae*. **(A)** Symptoms on the upper leaf surfaces. **(B)** Symptoms on the lower leaf surfaces. **(C)** Sporulating mycelium on the lower leaf surface.

**Figure 3:**
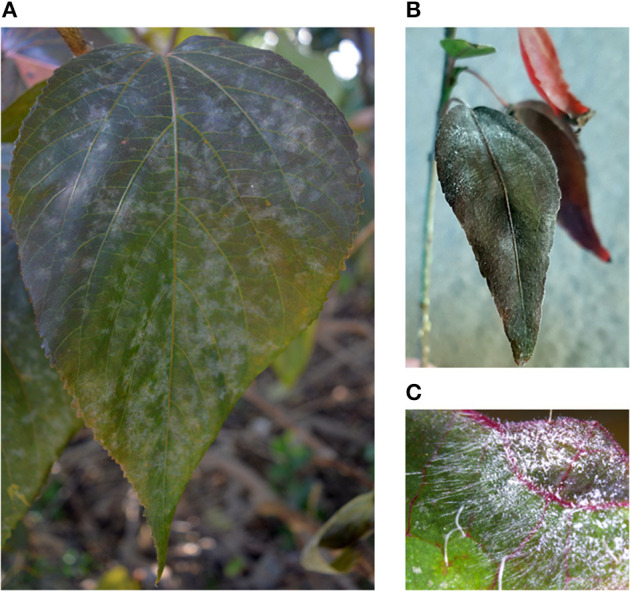
*Acalypha wilkesiana* infected with *Salmonomyces acalyphae*. **(A)** Symptoms on *A. wilkesiana* var. *macrophylla*. **(B)** Symptoms on *A. wilkesiana* cv. Inferno. **(C)** Sporulating mycelium on the upper leaf surface.

**Figure 4:**
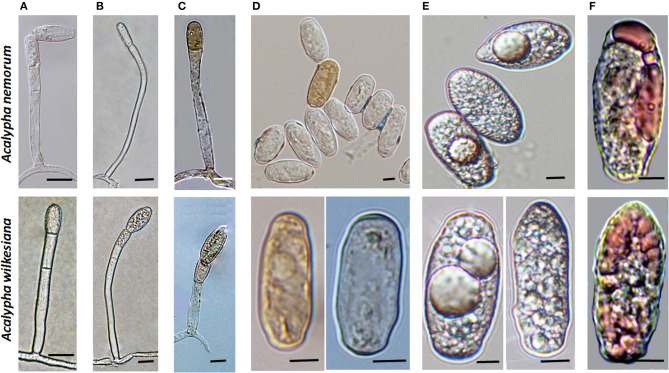
Conidiophores and conidia of *Salmonomyces acalyphae* on *Acalypha nemorum* and *A. wilkesiana* collected in Australia. **(A)** Short conidiophores. Bar = 15 μm. **(B)** Long conidiophores. Bar = 10 μm. **(C)** Melanized conidiophores. Bar = 10 μm. **(D)** Melanized and hyaline conidia after boiling in lactic acid. Note the disappearance of lipid inclusions due to boiling. Bar = 5 μm. **(E)** Fresh conidia filled with highly granular cytoplasm, occasionally containing voluminous oil droplets. Bar = 5 μm. **(F)** Conidia stained with Sudan Black B during gentle warming of the samples. A part of the lipid inclusions were stained, and deformed during warming. Bar = 5 μm.

**Figure 5:**
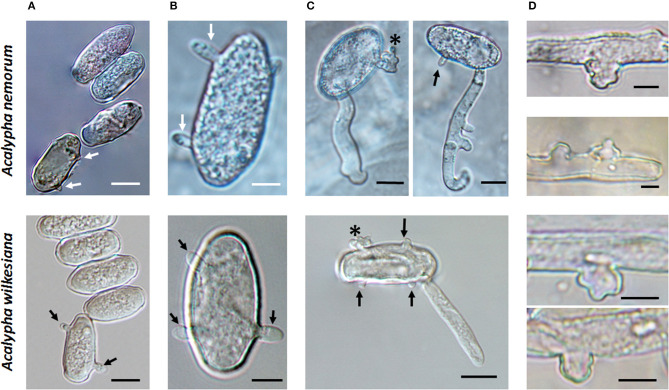
Germination of conidia of *Salmonomyces acalyphae* collected from *Acalypha nemorum* and *A. wilkesiana* on cellophane placed on Water Agar (WA) and onion scales (OS), respectively. Arrows indicate primary germ tubes, asterisks lobed secondary germ tubes. **(A)** Development of the first primary germ tube initials on WA. Bar = 10 μm. **(B)** More developed primary germ tubes on OS (above) and WA (below). Bar = 5 μm. **(C)** Secondary germ tubes with both simple and lobed ends developed on OS (above) and WA (below). Note the first hyphal elongation with lateral branching on OS. Bar = 10 μm. **(D)** Hyphal appressoria. Bar = 5 μm.

During the field surveys, powdery mildews with very unusual morphological characteristics were collected from *Acalypha nemorum* and *A. wilkesiana*. *Acalypha nemorum* is a native Australian shrub (Sagun et al., 2010), endemic to central and southern Queensland and north-eastern New South Wales, mostly in vine and open eucalypt forests (Forster, 1994). *Acalypha wilkesiana* is an ornamental shrub widely planted in subtropical and tropical regions of Australia and South East Asia (Sagun et al., 2010), and is native to Fiji (Sanz and Rodríguez, 2012). The symptoms of powdery mildew infections were unusual on *A. nemorum*, and mainly consisted of yellow spots on the upper leaf surfaces (Figure 2A), and intensively sporulating powdery mildew colonies on the corresponding lower leaf surfaces. The central, older parts of these colonies were brownish (Figures 2B,C). Occasionally, small, sporulating colonies were also observed on the upper surfaces of a few leaves. On *A. wilkesiana* var. *macrophylla* (Figure 3A) and cv. Inferno (Figures 3B,C), powdery mildew colonies covered large parts of both the upper and the lower leaf surfaces, causing typical symptoms of powdery mildew infections. The asexual morph exhibited some unusual characteristics on both *A. nemorum* and *A. wilkesiana*: (i) conidiophores were dimorphic, either short, Pseudoidium-like, that resembled common *Erysiphe* anamorphs (Figure 4A), or very long (Figure 4B); (ii) up to 3% of conidiophores and conidia were melanized (Figures 4C,D); (iii) conidia were filled with highly granular cytoplasm that appeared as a shiny, well-structured material, occasionally with voluminous oil droplets, when microscopically examined as fresh materials in water or lactic acid (Figure 4E); the lipid content of the granular cytoplasm and the droplets was confirmed by staining with Sudan Black B (Figure 4F); and (iv) germination of conidia, examined experimentally on sterile cellophane placed on water agar, and also on onion scales, differed from any conidial germination patterns described to date in the Erysiphales (Cook and Braun, 2009; Braun and Cook, 2012). In 24 to 48 h, germinated conidia, including the melanized ones, first produced short, thin, simple germ tubes (Figures 5A,B), up to four per conidium. Once developed, these primary germ tubes remained unchanged for 96 h. In 48 to 96 h, some conidia developed new, thicker secondary germ tubes that terminated in simple or lobed apices (Figure 5C). Some secondary germ tubes that had lobed to multi-lobed apices remained short, while others elongated, and became as long, or longer, than the conidium, ending in simple or lobed apices in 96 h (Figure 5C). Germination characteristics were similar on cellophane and onion scale surfaces. Hyphal appressoria were mostly lobed (Figure 5D). The unusual conidiophore and conidial morphology as well as the unusual conidial germination patterns indicated that this powdery mildew represents a genus on its own. Its molecular phylogenetic relationships were investigated based on nrDNA ITS, 18S and 28S sequences.

**Figure 6:**
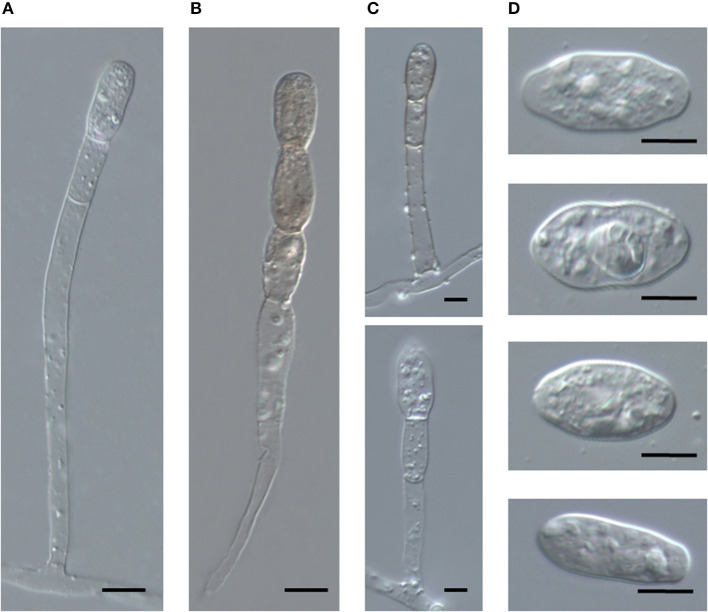
Conidiophores and conidia of *Salmonomyces acalyphae* on *Acalypha wilkesiana* collected in Argentina (HMJAU-PM91866), after boiling in lactic acid. **(A)** Long conidiophore producing conidia singly. Bar = 10 μm. **(B)** Conidiophore producing melanized conidia in a short chain. Bar = 10 μm. **(C)** Short conidiophores. Bars = 5 μm. **(D)** Conidia. Bar = 10 μm.

To obtain unambiguous ITS sequences of the powdery mildew on both *Acalypha* spp., the ITS regions of specimens BRIP 68804 and BRIP 68805, from *A. wilkesiana* and *A. nemorum*, respectively, were re-sequenced following cloning of the PCR products. This resulted in a total of 20 identical sequences which differed by a single nucleotide in the ITS2 region from those determined in four specimens (MUMH5149, MUMH5150, MUMH5152, and MUMH5559) collected from *A. wilkesiana* and *A. argentea* in Java, and described as *Pseudoidium javanicum* by Meeboon et al. (2013). The morphology (Figure 6) and the ITS sequence of a specimen collected from *A. wilkesiana* in Argentina (HMJAU-PM91866) were also identical to those of BRIP 68804 and BRIP 68805. However, according to Meeboon et al. (2013) the morphology of *Ps. javanicum* is very different from what we observed in our specimens from *A. nemorum* and *A. wilkesiana*. Most importantly, Meeboon et al. (2013) reported that all the conidiophores of *Ps. javanicum* were short and hyaline, typical of the asexual morphs of the genus *Erysiphe* (*Pseudoidium*), and its conidia were also described as typical of *Pseudoidium*. The conidial germination patterns were not described for *Ps. javanicum*.

**Figure 7:**
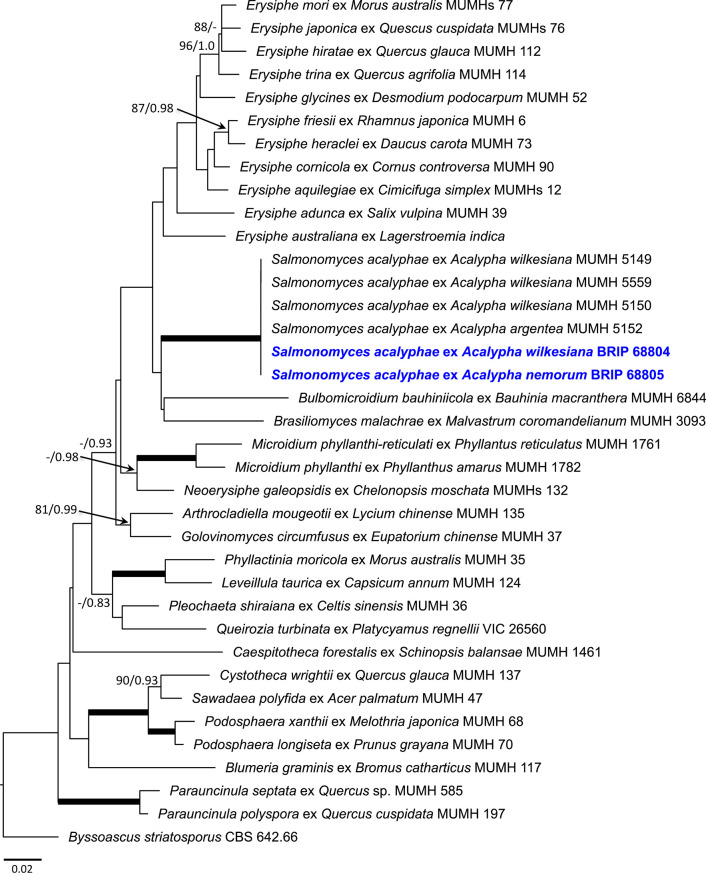
Maximum likelihood phylogram based on the concatenated sequences of the 28S, 5.8S, and 18S regions of the nuclear ribosomal DNA of representatives of all genera of the Erysiphales, except *Takamatsuella*. *Salmonomyces acalyphae* comb. nov. is shown in bold blue. The tree is rooted to *Byssoascus striatosporus* CBS 642.66. Maximum Likelihood bootstrap values >80% and Bayesian Posterior Probability values >0.80 are shown above or below the branches. Thickened branches represent Maximum Likelihood bootstrap value of 100% and Bayesian Posterior Probability of 1.00. The scale bar represents nucleotide substitutions per site.

The nrDNA 18S and 28S sequences were also determined in specimens BRIP 68804 and BRIP 68805, and analyzed together with the 18S, 28S, and 5.8S sequences of representative specimens of all but one powdery mildew genera accepted to date (Supplementary Table 1). *Takamatsuella* was the only genus of the Erysiphales that could not be included in this study as no authentic sequence data are available for this monotypic genus. The 28S and 5.8S sequences of *P. javanicum* determined by Meeboon et al. (2013) were included in the analysis, and the 18S sequences coded as missing, just like for *Brasiliomyces* (Supplementary Table 1). The dataset consisted of concatenated sequences of 36 taxa, the aligned matrix had a total length of 1,397 characters (28S: 663, 18S: 578, 5.8S: 156). The consensus phylogeny obtained using BI analysis supported the tree topology obtained with ML, therefore, only the best scoring ML phylogram is shown (Figure 7).

The Australian powdery mildew specimens from *A. nemorum* and *A. wilkesiana* had identical sequences at all the three loci. Their 28S and 5.8S sequences were also identical to those of MUMH5149, MUMH5150, MUMH5152, and MUMH5559, described as *P. javanicum* infecting *Acalypha* spp. (Meeboon et al., 2013). The clade consisting of the six specimens from *Acalypha* spp. had 100% Maximum Likelihood bootstrap (BS) value, and Bayesian Posterior Probability (PP) value of 1.0, being sister to a clade consisting of *Brasiliomyces malachrae* and *Bulbomicroidium bauhiniicola* (Figure 7). As both the morphological characteristics and the phylogenetic analysis have clearly indicated that the powdery mildew from *Acalypha* spp. is different from all the other powdery mildews, its allocation to a genus of its own was warranted.

Attempts to identify this powdery mildew led to the genus *Salmonomyces*, described by Chiddarwar (1959), as well as to *Erysiphe acalyphae* (≡ *Uncinula acalyphae*), a widespread species reported from various *Acalypha* spp. (Braun and Cook, 2012). The history of the taxonomic treatment of the *Acalypha* powdery mildew is summarized as follows. The first description of this powdery mildew dates back to Sawada (1943). He introduced the genus name *Orthochaeta* and the species name *O. acalyphae*, based on type material on *A. australis* from Taiwan, but both names are invalid, as they were published without Latin description or diagnosis (Art. 39.1). Tai (1946) published *Uncinula acalyphae*, based on a Chinese collection on *A. superba*, the first valid species name for this powdery mildew, which was later transferred to *Erysiphe* (Zheng and Chen, 1981). Doidge (1948) described *Uncinula eylesii* on *A. ciliata* from South Africa. Later, Chiddarwar (1959) introduced the new genus and species, *Salmonomyces kamatii*, for a powdery mildew found on *A. ciliata* in India. Chiddarwar (1959) treated this species as a member of a new genus, *Salmonomyces*, based on the unusual characteristics of the sexual morph, without being aware of the previous publications of Tai (1946) and Doidge (1948). Later, Pirozynski (1965) discussed the taxonomy of this *Acalypha* powdery mildew in detail, and reduced *Sa. kamatii* and *U. eylesii* to synonymy with *U. acalyphae*, based on re-examinations of type material of the synonyms involved, except for type material of *U. acalyphae*, which was not available for that study. Sathe (1969) considered *Salmonomyces* as a synonym of *Erysiphopsis* (Halsted, 1899), a North American genus with *Er. parnassiae* on *Parnassia caroliniana* as type species. Zheng and Chen (1981), Braun (1987), and Zheng and Yu (1987) followed Pirozynski (1965) taxonomic conclusions and synonymy. Braun and Cook (2012) treated this species under the name *Erysiphe acalyphae*. Recently, Meeboon et al. (2013) described *Pseudoidium javanicum* based on specimens collected from *Acalypha* spp. in Indonesia, and considered *Ps. javanicum* to be different from *E. acalyphae* by having shorter conidiophores.

**Figure 8:**
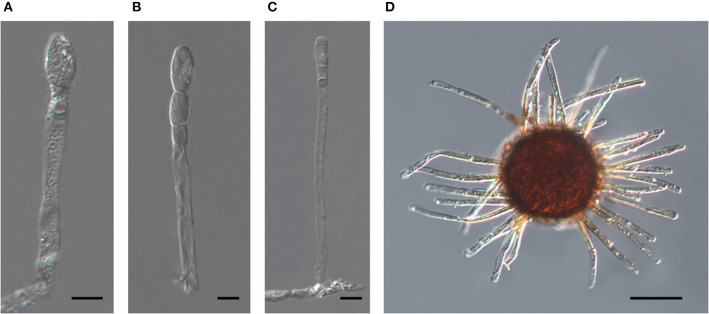
The asexual and the sexual morphs of *Salmonomyces acalyphae* on *Acalypha brachystachya* collected in China (HMAS 152), after boiling in lactic acid. **(A)** Short conidiophore producing conidia singly. Bar = 10 μm. **(B)** Conidiophore producing conidia in a short chain. Bar = 10 μm. **(C)** Long conidiophore producing conidia singly. Bar = 20 μm. **(D)** Chasmothecium. Bar = 50 μm.

The oldest validly introduced species name for the powdery mildew on *Acalypha* is *U. acalyphae*. The type specimen of *U. acalyphae* collected in South China (Tai, 1946), HMAS 153, and additional collections from China deposited at HMAS (Supplementary Table 1) were re-examined in this work. The characteristics of the asexual morph (Figures 8A–C) agreed with those of the specimens collected in Australia (Figures 4, 5) and Argentina (Figure 6); importantly, pigmented conidiophores and conidia have been observed in the type specimen and other Chinese specimens, as well. The sexual morph was also present in most of the HMAS specimens examined in this work (Figure 8D; Supplementary Table 1). Attempts to amplify nrDNA regions from the type specimen failed, and a trip to the type collection site in Southern China did not locate powdery mildew-infected *Acalypha* plants. The morphological agreement of the asexual morph of the type specimen of *U. acalyphae* with the specimens from Argentina and Australia, together with our phylogenetic analyses, warrant the introduction of the following new combination to accommodate *U. acalyphae*:

***Salmonomyces acalyphae***(F.L. Tai) L. Kiss, D.N. Jin and S.Y. Liu, **comb. nov**.

MycoBank, MB834807

Basionym: *Uncinula acalyphae* F.L. Tai, Bull. Torrey Bot. Club 73: 123, 1946.

≡ *Erysiphe acalyphae* (F.L. Tai) R.Y. Zheng and G.Q. Chen, Sydowia 34: 261, 1981.
= *Orthochaeta acalyphae* Sawada, Rep. Gov. Res. Inst. Formosa 85: 23, 1943, nom. inval. (Art. 39.1).
= *Uncinula eylesii* Doidge, Bothalia 4: 844, 1948 [type: South Africa, Zimbabwe, Harare (former Salisbury), on *Acalypha ciliata*, Feb. 1920, F. Eyles 2071 (PREM 13992, holotype)].
= *Salmonomyces kamatii* Chidd., Sydowia **13**: 56, 1959 [type: India, Poona, Purander Fort, on *Acalypha ciliata*, Sep. 1957, P.P. Chiddarwar 38 (HCIO, holotype; IMI83199, isotype)].
≡ *Erysiphopsis kamatii* (Chidd.) Sathe, Bull. Torrey Bot. Club 96: 102, 1969.
= *Pseudoidium javanicum* Meeboon and S. Takam., in Meeboon, Hidayat and Takamatsu, Mycoscience 54(3): 184, 2013 [types: Indonesia, West Java province, Bogor, Cibodas Botanical Garden, 7 Mar. 2012, on *Acalypha wilkesiana*, J. Meeboon et al. (TNS-F-46915, holotype; MUMH 5559, isotype)].

Illustrations: Tai (1946, p. 116, Figure 4), Zheng and Chen (198, p. 262, Figure 22), Braun (1987, p. 457, Pl. 207), Zheng and Yu (1987, p. 50, Figure 8), Braun and Cook (2012, p. 359, Figure 396).

References (descriptions and taxonomic discussions): Pirozynski (1965, p. 4–6), Braun (1987a, p. 234), Zheng and Yu (1987, p. 49).

Exsiccatae: Fungi Sinensis Exsiccati 4.

Holotype: on *Acalypha superba* [= *A. brachystachya*], China, Yunnan, Shishan, Kunming, Nov. 1938, F.L. Tai (HMAS 153). Isotypes: BPI 559117, HMAS 152.

Host range and distribution: on *Acalypha australis* [Taiwan (Amano, 1986), type of *Orthochaeta acalyphae*], *A*. ×*cristata* [Indonesia (Meeboon et al., 2013)], *A. indica* [India (Amano, 1986; Pande, 2008); Mauritius (Pirozynski, 1965; Amano, 1986), voucher specimen – IMI 102713], *A. ciliata* [India (Amano, 1986; Pande, 2008), voucher specimens – IMI 104267, 230417, 264545, type of *Salmonomyces kamatii*; South Africa (Pirozynski, 1965; Amano, 1986), type of *Uncinula eylesii*; Sudan (Amano, 1986); *Zambia* (Amano, 1986); Zimbabwe (Amano, 1986)], A. lanceolata [India (Amano, 1986), voucher specimen – IMI 90203], *A. nemorum* [Australia, this publication], *A. sinensis* [Tanzania (Amano, 1986)], *A. superba* [China (Tai, 1946; Amano, 1986; Zheng and Yu, 1987), type of *Uncinula acalyphae*; India (Amano, 1986; Pande, 2008); Uganda (Amano, 1986)], *A. wilkesiana* [Argentina (this publication); Australia (this publication); Indonesia (Meeboon et al., 2013), type of *Pseudoidium javanicum*], *Acalypha* sp. [Sudan, voucher specimen – IMI 45223; Zambia (Amano, 1986), voucher specimen – IMI 87889, 95790], *Euphorbiaceae*.

Notes: Meeboon et al. (2013) reported *Pseudoidium javanicum* on “*Acalypha argentea* hort.,” which does not exist as a validly published name, therefore, this host can only be referred to as *Acalypha* sp.

Morphology

Mycelium and asexual morph (based on the examination of collections from Australia, Argentina, and Indonesia and a re-examination of type material of *Uncinula acalyphae*): Mycelium amphigenous, thin, effuse, persistent, rarely evanescent; hyphae branched, straight to sinuous, thin-walled, smooth, hyaline; hyphal appressoria nipple-shaped or slightly to distinctly lobed, single or in opposite pairs; conidiophores dimorphic, short, and Pseudoidium-like, consisting of a 25–75 × 5–7 μm foot-cell, followed by 0–2 shorter cells, each (9–)12–20(−24) × 6–8 μm, and a single conidium or conidium initial, and/or long and slender, consisting of a very long foot-cell, 50–150(−243) × 4.5–7 μm, followed by 0–3 shorter cells, each (11–)16–23(−29) × 6–8 μm, and a single conidium or conidium initial or conidia formed in short chains, most conidiophores colorless, but up to three percent becoming pigmented; conidia ellipsoid-ovoid, subcylindrical to doliiform, occasionally with irregular margins, or pointed at one end, 16–32 × 8–16 μm, hyaline or up to three percent becoming pigmented, brownish, content (cytoplasm) granular, fresh conidia with voluminous, up to 9 μm diameter oil droplets.

Conidial germination (based on Australian collections): Primary germ tubes short, thin, simple germ tubes, up to four per conidium. Secondary germ tubes thicker, very short, or as long, or longer, than the conidium, ending in simple or lobed apices.

Sexual morph (based on the holotype, HMAS 153, and the isotype, HMAS 152): Chasmothecia scattered to gregarious, (70–)80–145 μm diam.; peridium cells irregularly polygonal to rounded, not very conspicuous, 5–20 μm diam.; appendages numerous, (10–)20–50, arising equatorially or from the upper half of the ascomata, rather stiff, setiform, 0.5–1.5 times as long as the chasmothecial diam., 4–9 μm wide, width uniform or decreasing from base to top, apex straight, pointed to rounded, rarely somewhat inflated, somewhat recurved or irregular, rarely abruptly constricted just below the apex or at the base, 0–1-septate, occasionally few septum-like lines in the upper half, pigmented, brown throughout or only below, paler toward the tip, apex hyaline or yellowish, walls thin to moderately thick, rarely thin above and thick toward the base, smooth to verrucose; asci 3–12, ellipsoid-obovoid, 30–60 × 20–35 μm, sessile or short-stalked, mostly immature with undeveloped ascospores (4 per ascus, 11–17 × 8–14 μm, according to Tai, 1946).

### Detection of an Uncultured Fungus From *Rhododendron* Powdery Mildew Samples

In most cases, the nested PCRs that included the powdery mildew-specific PMITS1/PMITS2 primer pair (Cunnington et al., 2003) resulted in ITS sequences that were identical, or at least 98% similar to those of different powdery mildews as revealed by BLAST searches in GenBank. Surprisingly, these PCRs, when performed with DNA samples from any powdery mildew colonies sampled from *Rhododendron* spp., consistently yielded ITS sequences that were identical or over 97% similar to those of several other, unidentified and uncultured fungi. Those fungal ITS sequences were reported from *Holcus lanatus* roots collected in Germany (JF755916, Kreyling et al., 2012), house dust and indoor air samples collected in the USA (KF800177, Rittenour et al., 2014), *Fagus sylvatica* leaf litter collected in Austria (JF495199), and rainwater in China (KX516515, KX516323, and KX516237). These results indicate that some ubiquitous, cosmopolitan, but so far unidentified fungi were always associated with the powdery mildew colonies sampled from *Rhododendron* spp. in different places in this study (Table 2), and their ITS region was amplified consistently with the PMITS1/PMITS2 primer pair. These were the only powdery mildew samples in this work where the nested PCR protocol (Cunnington et al., 2003) has never amplified the ITS region of the respective powdery mildew fungus. A representative sequence obtained from *Rhododendron* samples was deposited in GenBank as the ITS sequence of an uncultured fungus under accession number MT152274.

### Sequencing of an ITS Fragment of *E. izuensis*

**Figure 9:**
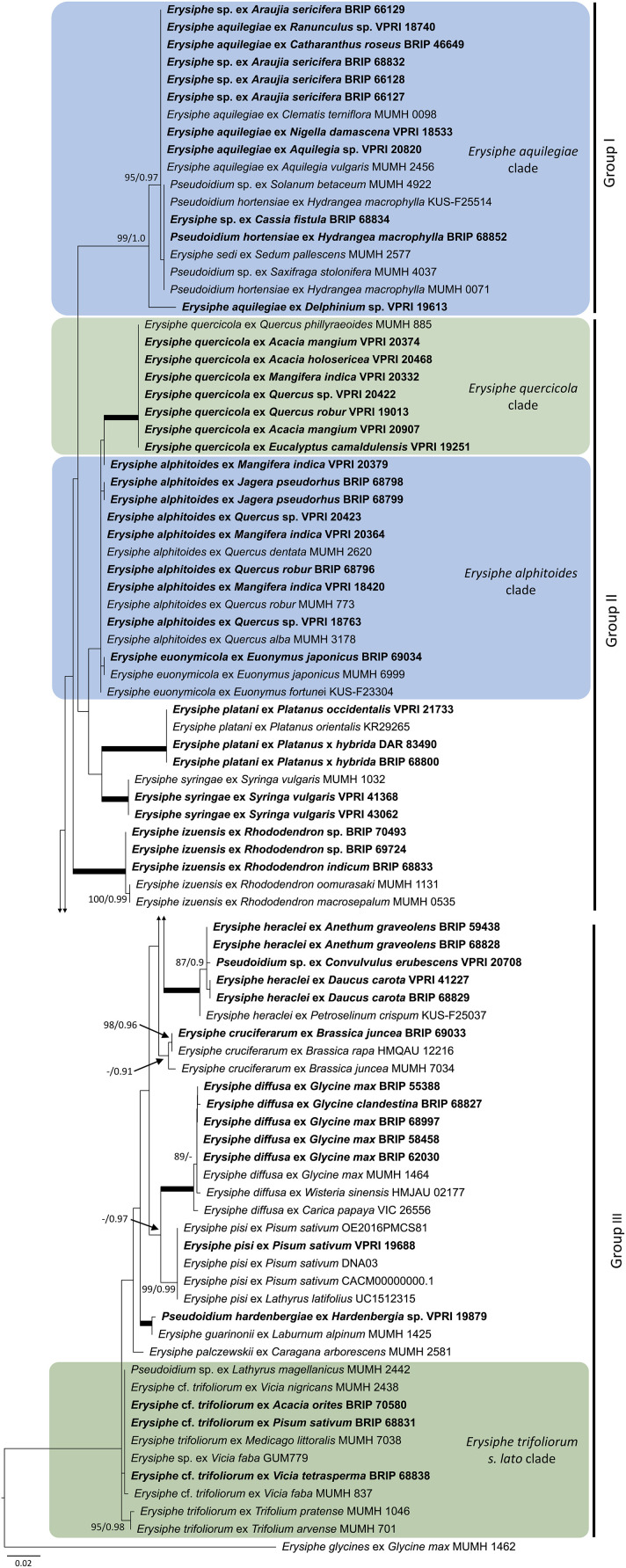
Maximum likelihood phylogram based on the internal transcribed spacers of the nuclear ribosomal DNA and the intervening 5.8S region for powdery mildew species belonging to the *Microsphaera* lineage of the genus *Erysiphe* (Takamatsu et al., 2015a). Groups I to III represent the clades defined by Takamatsu et al. (2015a). The tip labels in bold represent specimens collected in Australia (supporting data in Table 2). All the other specimens were collected overseas (supporting data in Supplementary Table 3). The tree is rooted to *Erysiphe glycines* MUMH 1462. Maximum Likelihood bootstrap values >80% and Bayesian Posterior Probability values >0.80 are shown above or below the branches. Thickened branches represent Maximum Likelihood bootstrap value of 100% and Bayesian Posterior Probability of 1.00. The scale bar represents nucleotide substitutions per site.

As the powdery mildew species collected from *Rhododendron* was identified as *E. izuensis* based on the morphology of the asexual and sexual morphs (Figure 1A), a species-specific primer pair was designed (Table 1) and used to amplify and sequence an ~450 bp long fragment of the ITS region of *E. izuensis* in six specimens. These were all identical to the corresponding ITS region of an *E. izuensis* specimen from Japan (LC009975), and were included in the phylogenetic analysis of the *Microsphaera* lineage of *Erysiphe* (Figure 9).

### Species Belonging to the *Uncinula* Lineage of the Genus *Erysiphe*

The genus *Erysiphe* includes the highest number of species within the Erysiphales (Braun and Cook, 2012). Two major lineages, *Microsphaera* and *Uncinula*, have been repeatedly identified within the genus (Takamatsu et al., 2015a,b). The ITS sequences of these two groups are too divergent to be included in a single analysis (Takamatsu et al., 2015a,b). As the *Uncinula* lineage is only represented by two species in this study, *E. necator* and *E. australiana* (Table 2), no phylogenetic analysis was performed for this group.

### Species Belonging to the *Microsphaera* Lineage of the Genus *Erysiphe*

Based on morphological examinations and ITS sequence analyses, a total of 51 specimens collected in this study or reported from Australia earlier (Table 2) belonged to the *Microsphaera* lineage. These specimens represented 15 species. Their ITS sequences were analyzed together with 31 ITS sequences from similar taxa collected overseas (Supplementary Table 3). The consensus phylogeny (Figure 9) revealed that powdery mildew infections of a number of native plants were caused by common species known from overseas. For example, the severe powdery mildew infections of young individuals of a native rainforest tree, *Jagera pseudorhus* (Sapindaceae) (Supplementary Figure 1A), recorded in two distant locations in Queensland (Table 2), was caused by *E. alphitoides*. This powdery mildew is a well-known pathogen of oaks (*Quercus* spp.), and has also been found on other woody hosts such as mango (*Mangifera indica*), and on herbaceous hosts, such as *Oenothera* spp., in different parts of the world (Limkaisang et al., 2006; Takamatsu et al., 2007, 2015a; Bereczky et al., 2015; Desprez-Loustau et al., 2017, 2018). In Australia, *E. alphitoides* was detected earlier on *Quercus* spp. and *M. indica* (Table 2). The ITS sequences determined in both specimens collected from *J. pseudorhus* were identical, and differed in one nucleotide in the ITS2 region from *E. alphitoides* specimens collected in Australia (Figure 9). *Erysiphe euonymicola*, recorded for the first time in Australia based on molecular evidence, is closely related to *E. alphitoides* (Figure 9).

*Erysiphe quercicola* is another species that infects *Quercus* spp., and also a number of distantly related woody plants, such as *Citrus* spp., *M. indica*, and rubber tree (*Hevea brasiliensis*), in different parts of the world (Takamatsu et al., 2007; Desprez-Loustau et al., 2017, 2018). It was recorded earlier in Australia on *M. indica*, and also on the native *Acacia holosericea, A. mangium*, and *Eucalyptus camaldulensis* (Table 2). No information is available about the severity of the disease caused by *E. quercicola* on any of these plants in Australia. During our study, powdery mildew infection was observed on another native *Acacia* species, *A. orites*, where the mycelium was restricted to the bipinnate leaves at the tips of the phyllodes of young plants (Supplementary Figure 1B). Based on the ITS sequence analysis, the causal agent belonged to the *E. trifoliorum* s. lato group (Figure 9). Specimens with ITS sequences identical to those of the *A. orites* powdery mildew were also collected from pea (*Pisum sativum*) and *Vicia tetrasperma* in this study, and the same ITS sequences were available in GenBank for a number of overseas powdery mildews (Figure 9). The taxonomy of the *E. trifoliorum* s. lato group is still unresolved.

Powdery mildews belonging to the *E. aquilegiae* group, defined by Takamatsu et al. (2015a), have been reported from plant species of at least 15 families in different parts of the world (Jankovics et al., 2008; Meeboon and Takamatsu, 2015), including Australia (Cunnington et al., 2004a; Liberato and Cunnington, 2006; Southwell et al., 2018). This study confirmed that a powdery mildew on *Araujia sericifera* reported recently from Australia by Southwell et al. (2018) belongs to this group based on its ITS sequence. A similar powdery mildew infecting *Cassia fistula* has also been identified in this work (Figure 9). To date, *Ps. cassiae-siameae* is the only known *Erysiphe* species from *C. fistula*, reported from Africa and Asia (Braun and Cook, 2012), but ITS sequences are not available for this taxon. The ITS sequences determined in our specimen from *C. fistula* were identical to a number of other powdery mildews reported from overseas, and also to *P. hortensiae* infecting *Hydrangea macrophylla* (Figure 9). This is the first confirmation of the latter species in Australia. Clearly, the taxa belonging to the *E. aquilegiae* group based on their ITS sequences need taxonomic treatment (Shin et al., 2019).

### *Golovinomyces* Species

**Figure 10:**
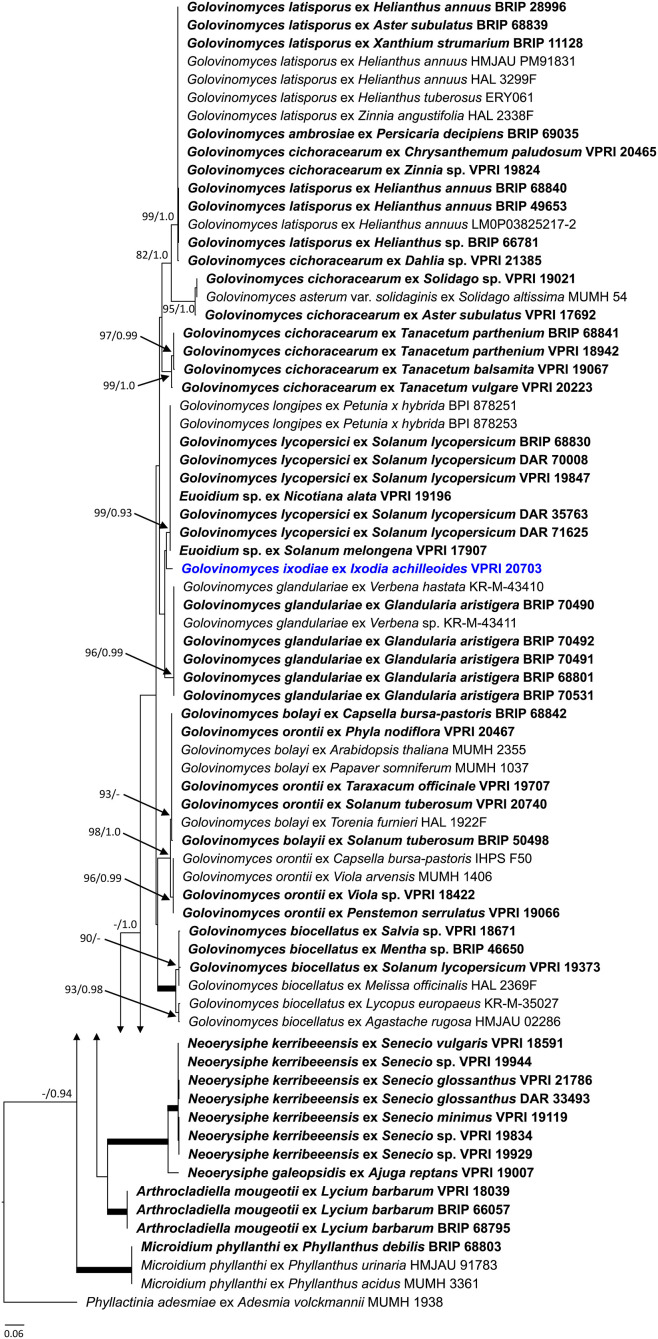
Maximum likelihood phylogram based on the internal transcribed spacers of the nuclear ribosomal DNA and the intervening 5.8S region for powdery mildew species belonging to *Arthrocladiella, Golovinomyces, Microidium*, and *Neoerysiphe* genera. The tip labels in bold represent specimens collected in Australia (supporting data in Table 2). All the other specimens were collected overseas (supporting data in Supplementary Table 3). *Golovinomyces ixodiae* comb. nov. is shown in bold blue. The tree is rooted to *Phyllactinia adesmiae* MUMH 1938. Maximum Likelihood bootstrap values >80% and Bayesian Posterior Probability values >0.80 are shown above or below the branches. Thickened branches represent Maximum Likelihood bootstrap value of 100% and Bayesian Posterior Probability of 1.00. The scale bar represents nucleotide substitutions per site.

Based on morphology and ITS sequence analyses, a total of 10 *Golovinomyces* species have been identified in Australia (Table 2; Figure 10). Two of these, *G. bolayi* and *G. glandulariae* have only been recently described. *Golovinomyces bolayi* was delimited from the *G. orontii* species complex, and recognized as being globally distributed on a wide range of host plant families (Braun et al., 2019). This newly described species was represented by two specimens examined in this study, collected from *Capsella bursa-pastoris* and *Solanum tuberosum*. Other Australian specimens from *S. tuberosum, Taraxacum officinale*, and *Phyla nodiflora*, identified earlier as *G. orontii* (Cunnington et al., 2010), had ITS sequences identical to the Australian and overseas *G. bolayi* specimens (Figure 10). Many other *G. orontii* specimens with ITS sequences identical to all these powdery mildews have been reported earlier from a wide range of host plants across the globe; these data may indicate the polyphagous nature of these fungi (Matsuda and Takamatsu, 2003; Takamatsu et al., 2013; Braun et al., 2019).

The other recently recognized species, *G. glandulariae*, was described from Australia as a pathogen of *Glandularia aristigera* [= *Verbena aristigera*] (Crous et al., 2019), a verbenaceous species native to South America (O'Leary and Thode, 2016). It seems that this is not an endemic Australian powdery mildew species either because the ITS sequences determined in all the *G. glandulariae* specimens were identical to two other *Golovinomyces* specimens, KR-M-43410 and KR-M-43411 (GenBank acc. nos.: LC076839 and LC076840, respectively), collected from *Verbena* in Germany without being identified at the species level (Scholler et al., 2016). In addition, a powdery mildew identified as *G. verbenae* without DNA sequence data has been recently recorded in Europe on *G. aristigera* (Kruse et al., 2018).

*Ixodia achillaeoides* was the only Australian native hosting a *Golovinomyces* species. This was described as *Oidium ixodiae* by Cunnington et al. (2005a), and later transferred to *Euoidium* by Braun and Cook (2012). As of February 2020, there are no powdery mildew records in GenBank with ITS sequences identical to that of *E. ixodiae*. Based on the current “International Code of Nomenclature for algae, fungi, and plants (Shenzhen Code, 2018),” and the “One fungus one name” principle, *Euoidium* being a heterotypic synonym of *Golovinomyces*, all phylogenetically and taxonomically confirmed *Euoidium* spp. have to be transferred to *Golovinomyces* (Braun, 2012, 2013):

***Golovinomyces ixodiae***(Cunningt., Beilharz and Pascoe) U. Braun and L. Kiss, **comb. nov**.

MycoBank, MB834808

Basionym: *Oidium ixodiae* Cunningt., Beilharz and Pascoe, Australas. Pl. Pathol. 34(1): 92, 2005.

= *Euoidium ixodiae* (Cunningt., Beilharz and Pascoe) U. Braun and R.T.A. Cook, Taxonomic Manual of the Erysiphales (Powdery Mildews): 334, 2012.

*Golovinomyces ixodiae* is sister to the clade containing *G. longipes, G. lycopersici* and *Euoidium* sp. on *Solanum melongena* and *Nicotiana alata* with identical ITS sequences (Cunnington et al., 2005b) (Figure 10). Amongst these taxa, *G. lycopersici* on tomato has never been recorded outside Australia (Kiss et al., 2005; Braun et al., 2019), although tomato is a crop introduced from overseas. In Australia, *G. lycopersici* has been reported since 1980 (Kiss et al., 2001), including in this study (Table 2). This species is morphologically and molecularly similar to *G. longipes* (Cunnington et al., 2005a; Kiss et al., 2008; Kovács et al., 2011) that has caused epidemics on solanaceous vegetables and ornamentals in Europe and the USA (Kiss et al., 2008).

All the powdery mildew specimens collected from sunflower (*Helianthus annuus*) in this study were identified as *G. latisporus* based on both ITS sequence analyses (Figure 10) and morphological characteristics, particularly the unique conidial germination patterns (Cook and Braun, 2009; Braun and Cook, 2012). This species was delimited from the *G. cichoracearum* complex based on its morphology (Cook and Braun, 2009). A number of Australian and overseas specimens identified earlier as *G. cichoracearum* had ITS sequences identical to *G. latisporus* (Figure 10). Another globally distributed species separated from the *G. cichoracearum* complex is *G. ambrosiae*, characterized with an ITS sequence that is identical to that of *G. latisporus*, but with morphological characteristics that readily distinguish it from the latter species (Braun et al., 2019). A recent multi-locus analysis distinguished *G. latisporus* and *G. ambrosiae* based on non-nrDNA sequences (Qiu et al., 2020). In Australia, *G. ambrosiae* was identified on *Persicaria decipiens*, the first member of the Polygonaceae that has been reported as a host of a *Golovinomyces* species worldwide (Braun et al., 2019).

### *Neoerysiphe, Arthrocladiella*, and *Microidium* Species

As the alignments of the ITS sequences of the species of *Golovinomyces, Neoerysiphe, Arthrocladiella*, and *Microidium* were non-ambiguous, these were analyzed together. The results for the latter three genera are shown in Figure 10, and are discussed below, separately from *Golovinomyces*, for clarity purposes.

Powdery mildews representing the genus *Neoerysiphe* were not found in this study, but have been collected and intensively studied earlier in Australia (Cunnington et al., 2003; Beilharz et al., 2010). One of these fungi, named as *N. kerribeeensis*, produced chasmothecia on *S. glossanthus*, and was described as the first teleomorphic powdery mildew recognized on a native host in Australia (Beilharz et al., 2010). Its asexual morph has also been identified on several other *Senecio* spp. introduced to Australia (Beilharz et al., 2010). As of February 2020, there are no powdery mildew records in GenBank with ITS sequences identical or at least 98% similar to that of *N. kerribeeensis*. The closest taxon is *N. galeopsidis* (Figure 10), which has been recorded in Australia (Cunnington et al., 2003), and is known from other parts of the world as well (Takamatsu et al., 2008; Heluta et al., 2010).

*Arthrocladiella mougeotii*, representing a monotypic genus, has been consistently found on its host, *Lycium barbarum*, an introduced solanaceous species, in Australia (Cunnington et al., 2003; Kiss et al., 2018a). This species is found globally on *Lycium* spp. (Braun and Cook, 2012). In contrast, *Microidium phyllanthi*, one of the four described *Microidium* species (To-anun et al., 2005; Meeboon and Takamatsu, 2017), is reported here for the first time from Australia (Table 2).

### *Podosphaera* and *Sawadaea* species

**Figure 11:**
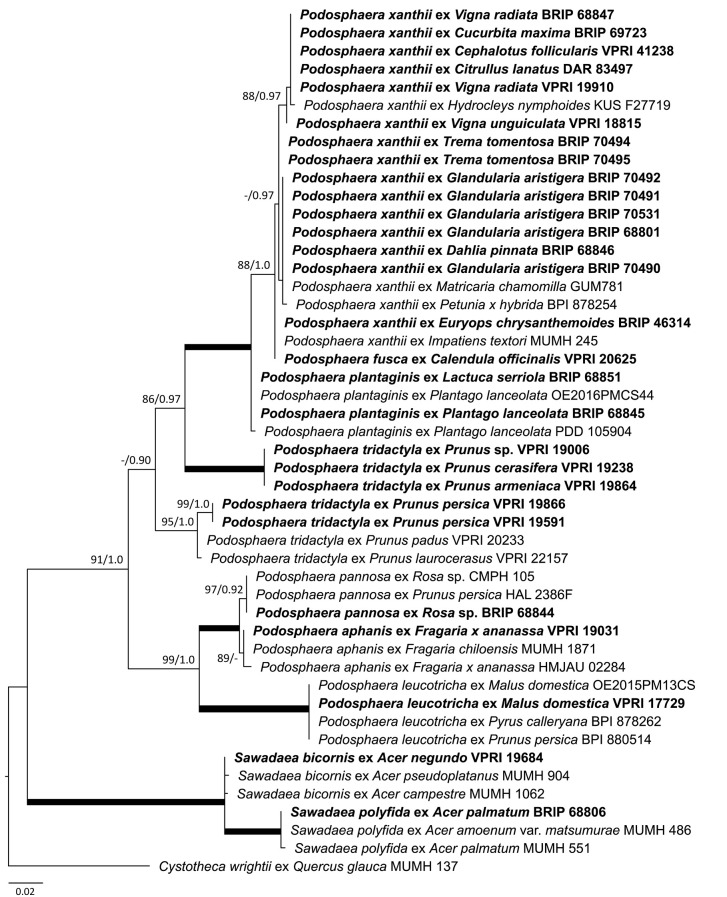
Maximum likelihood phylogram based on the internal transcribed spacers of the nuclear ribosomal DNA and the intervening 5.8S region for powdery mildew species belonging to *Podosphaera* and *Sawadaea* genera. The tip labels in bold represent specimens collected in Australia (supporting data in Table 2). All the other specimens were collected overseas (supporting data in Supplementary Table 3). The tree is rooted to *Cystotheca wrightii* MUMH 137. Maximum Likelihood bootstrap values >80% and Bayesian Posterior Probability values >0.80 are shown above or below the branches. Thickened branches represent Maximum Likelihood bootstrap value of 100% and Bayesian Posterior Probability of 1.00. The scale bar represents nucleotide substitutions per site.

This study confirmed the presence of seven *Podosphaera* species in Australia (Figure 11; Table 2), all known to be globally distributed on their hosts (Braun and Cook, 2012). Amongst these taxa, *P. xanthii* is regarded as a heterogeneous species complex of many races that could each be specialized to distinct hosts or have broad host ranges that include cucurbits, composites, legumes, and several other plant families (Hirata et al., 2000; Braun et al., 2001). In Australia, *P. xanthii* has been identified on nine plant species (Table 2); some of these hosts are only distantly related. For example, powdery mildew infections observed in this study on young leaves of *Trema tomentosa* (Cannabaceae), a native rainforest tree species, were caused by *P. xanthii* (Supplementary Figure 1C). These infections, observed on a single site in Queensland, in 2 consecutive years (Table 2), are of interest because *T. tomentosa* is reported for the first time as a powdery mildew host, and also because *P. xanthii*, despite its broad host range, is uncommon on woody species. Earlier, another interesting Australian record of *P. xanthii* came from a nursery where potted *Cephalotus follicularis* (Cephalotaceae) plants became heavily infected with this powdery mildew (Cunnington et al., 2008a). This unusual host of *P. xanthii* is an attractive insectivorous plant native, and endemic to Western Australia. A number of its clones have been propagated commercially in different parts of the world for decades (Ko et al., 2010); therefore, these may have been repeatedly exposed to powdery mildews under various conditions. However, to our knowledge, the only powdery mildew that has been recorded on this native plant so far was *P. xanthii* in a nursery in Melbourne (Cunnington et al., 2008a). In nurseries, greenhouses and other artificial environments, *P. xanthii* has caused a number of unusual and unique powdery mildew infections, such as a serious disease of jellyfish tree (*Medusagyne oppositifolia*), imported from the Seychelles to the United Kingdom (Pettitt et al., 2010), or an infection of waterpoppy (*Hydrocleys nymphoides*), a monocot grown in pots in an urban area in Korea (Cho et al., 2018).

*Podosphaera plantaginis* infecting *Plantago lanceolata* in many parts of the world has become a model species in evolutionary and ecological studies of wild plant-pathogen interactions (Numminen et al., 2019). Its presence in Australia is confirmed in this study. Mating assays and molecular evidence have demonstrated that *P. plantaginis* is homothallic (Tollenaere and Laine, 2013), and therefore it is not surprising that its chasmothecia have been found on *Pl. lanceolata* (Figure 1B). Interestingly, *P. plantaginis* was also identified on *Lactuca serriola* in Western Australia (Figure 11; Table 2). This is the first record of *P. plantaginis* on this host. Another powdery mildew species, *G. bolayi* (earlier known as *G. cichoracearum* s. lat.), has been commonly found on *L. serriola* in many parts of the globe (Lebeda and Mieslerová, 2011; Braun et al., 2019), but was not recorded on *L. serriola* in Australia.

Powdery mildews infecting *Prunus* spp. were intensively studied by Cunnington et al. (2005c) based on specimens collected in Australia and elsewhere. Recently, the taxonomy of this group was revised (Moparthi et al., 2019; Meeboon et al., 2020), therefore the identity of the Australian specimens should be revisited.

Powdery mildew infections of maples (*Acer* spp.) are caused by species belonging to the genus *Sawadaea* (Hirose et al., 2005). So far, the presence of two species, *S. bicornis* and *S. polyfida*, have been confirmed in Australia (Figure 11; Table 2).

### *Blumeria* and *Leveillula* Species

**Figure 12:**
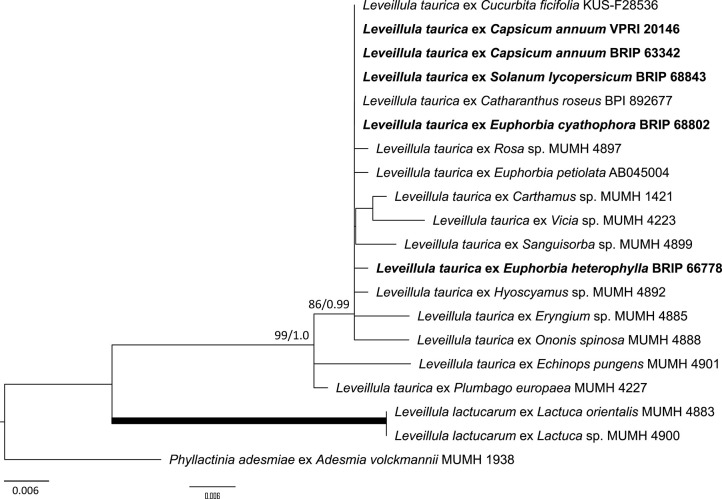
Maximum likelihood phylogram based on the internal transcribed spacers of the nuclear ribosomal DNA and the intervening 5.8S region for powdery mildew species belonging to the genus *Leveillula*. The tip labels in bold represent specimens collected in Australia (supporting data in Table 2). All the other specimens were collected overseas (supporting data in Supplementary Table 3). The tree is rooted to *Phyllactinia adesmiae* MUMH 1938. Maximum Likelihood bootstrap values >80% and Bayesian Posterior Probability values >0.80 are shown above or below the branches. Thickened branches represent Maximum Likelihood bootstrap value of 100% and Bayesian Posterior Probability of 1.00. The scale bar represents nucleotide substitutions per site.

Both genera include a number of distinct phylogenetic lineages (Inuma et al., 2007; Khodaparast et al., 2007, 2012; Kusch et al., 2020) that have not been taxonomically resolved. These powdery mildew lineages represent globally distributed species complexes, e.g., *B. graminis* on cereals and wild grasses; and *L. taurica* on a wide range of vegetables, ornamentals, and wild plant species. *Blumeria graminis* occurs on all major cereal species in Australia (Simmonds, 1966; Golzar et al., 2016), but has been confirmed molecularly only on barley (*Hordeum vulgare*) and *Phalaris canariensis* (Table 2). *Leveillula taurica* has been identified on *Capsicum* (Cunnington et al., 2003), tomato (*Solanum lycopersicum*) and two species of *Euphorbia* in Australia by morphology and ITS sequence analyses (Figure 12; Table 2).

### Number of Powdery Mildew Species in Australia and Overseas

The number of powdery mildew species in the genera *Erysiphe, Golovinomyces, Neoerysiphe*, and *Podosphaera*, reported for Asia, Europe, and North America (Braun and Cook, 2012), were compared with those recorded in Australia in this study. The data in Braun and Cook (2012) may be biased by doubtful species, species complexes, collection intensity, and other factors, whereas the data from this study were similarly biased by collection intensity. The comparison showed that the number of species of *Erysiphe, Golovinomyces, Neoerysiphe*, and *Podosphaera*, were much lower in Australia than the other continents (Table 3).

**Table 3:** Number of powdery mildew species belonging to four genera in Australia, based on this paper, and in Asia, Europe and North America, compiled from Braun and Cook (2012).

**Genus**	**Australia**	**Asia**	**Europe**	**North America**
*Erysiphe*	17	340	103	100
*Podosphaera*	7	85	50	40
*Golovinomyces*	10	37	33	34
*Neoerysiphe*	2	7	5	3

## Discussion

The establishment of the first European settlement in Australia in 1788 represents a sharp biogeographic watershed in the history of the entire Australian flora. There is no other continent where the introduction and spread of European agricultural and forestry practices, including the cultivation of crops imported from overseas, had happened as suddenly, and as recently as in Australia (Fensham and Laffineur, 2019). Almost all broad-acre, fruit, and vegetable crops currently grown in Australia were recently introduced from overseas (Kirkpatrick, 1999; Randall, 2007). In addition, the Acclimatization Societies founded in different part of Australia in the nineteenth and early twentieth century to “enrich” the flora and fauna in Australia, and create landscapes that were more familiar for the European settlers (Cook and Dias, 2006) had an enormous contribution to the introduction and establishment of a high number of ornamental and other plant species from all over the world, together with the associated microbiota. Currently, the number of vascular plant species introduced to Australia since 1788 outnumbers the native species; the entire native Australian vegetation has been deeply transformed by massive land management practices and human-mediated plant introductions (Kirkpatrick, 1999; Randall, 2007).

During our investigation started in 2017, most powdery mildews were collected from introduced hosts. In this study, powdery mildew was found on only four native plants, i.e., *Acacia orites, Acalypha nemorum, Jagera pseudorhus*, and *Trema tomentosa*. Each of these native species was infected with a single powdery mildew species, i.e., *Erysiphe* cf. *trifoliorum, Salmonomyces acalyphae, E. alphitoides*, and *Podosphaera xanthii*, respectively, known to infect a number of other plant species overseas (Braun and Cook, 2012; Takamatsu et al., 2015a; Desprez-Loustau et al., 2017). These four powdery mildew species were also identified in this study on introduced hosts; for example on *Pisum sativum, Acalypha wilkesiana, Quercus robur*, and *Vigna* spp., respectively. All powdery mildew infections of the four natives were collected in dry rainforests. On *A. orites*, the infection was limited to the bipinnate leaves of young plants. As well, only the young leaves of *T. tomentosa* were infected with powdery mildew, while on the other two natives, *A. nemorum* and *J. pseudorhus*, the infection was widespread on older leaf tissues. These four native plants may be considered as “accidental” hosts of the respective powdery mildews, although *E. alphitoides* was found in two distant locations on *J. pseudorhus*, and both *P. xanthii* and *S. acalyphae* have been repeatedly identified in the same site, but on different plants during surveys in 2018 and 2019. These observations suggest that the respective powdery mildews have been established on their native hosts. Less is known about powdery mildew infections of other Australian native species belonging to the genera *Acacia, Cephalotus, Convolvulus, Eucalyptus, Hardenbergia, Ixodia*, and *Senecio*, reported prior to this study (Cunnington et al., 2003, 2004b, 2005a, 2008a; Limkaisang et al., 2006; Beilharz et al., 2010). With the notable exception of G. *ixodiae* (= *Oidium ixodiae*) on *I. achillaeoides* (Cunnington et al., 2005a), and *N. kerribeeensis* on *S. glossanthus* (Beilharz et al., 2010), to be discussed below, all the other powdery mildews recorded on these natives are known as polyphagous species, i.e., infecting diverse other plant species, both in Australia and overseas. Therefore, in this paper we hypothesize that all powdery mildews have been introduced to Australia, since 1788, and some of these have adapted to become pathogens of a few native plant species, as a result of host range expansion events. Our arguments for this hypothesis are presented below.

Host range expansions appear to be frequent events in the evolution of some powdery mildews (Vági et al., 2007; Kirschner, 2010; Jankovics et al., 2011; Takamatsu, 2013a; Menardo et al., 2016; Beenken, 2017; Frantzeskakis et al., 2019a), and these could explain a number of “accidental” hosts of these fungi in different parts of the world. The genetic and genomic background of such rapid evolutionary processes has been partly revealed in obligate biotrophic plant pathogens (for reviews, see Frantzeskakis et al., 2019a; Thines, 2019). These events may explain the quick establishment of some polyphagous species, notably *P. xanthii, E. alphitoides, E. quercicola, E*. cf. *trifoliorum*, and *P. xanthii*, on Australian native plants. A number of unexpected records of this study, i.e., powdery mildew species on introduced plants that have not been known as their hosts overseas, including *Erysiphe* sp. (in the *E. aquilegiae* complex) on *C. fistula, G. ambrosiae* on *Pe. decipiens*, or *P. plantaginis* on *La. serriola*, may have resulted from rapid host range expansion events that have recently happened in the deeply disturbed Australian vegetation.

In this study, all powdery mildews were identified by morphology and ITS sequences, supplemented with 18S and 28S sequence analyses in the case of *Salmonomyces*. The use of ITS as the single DNA barcode for species identifications is clearly a bias in powdery mildew research (Kovács et al., 2011; Ellingham et al., 2019), and the limits of the routine fungal identification procedures based on ITS sequences are well-known in mycology (Nilsson et al., 2006; Schoch et al., 2012; Kiss, 2012; Nilsson et al., 2014; Irinyi et al., 2015; Hibbett et al., 2016; Selosse et al., 2016). ITS sequences are currently still the only reliable molecular tool for powdery mildew species identifications, despite recent advancements toward new DNA barcodes (Desprez-Loustau et al., 2017; Ellingham et al., 2019). These could be boosted by the recently determined genomes of distinct lineages (Bindschedler et al., 2016; Frantzeskakis et al., 2019b; Bradshaw and Tobin, 2020). However, closely related powdery mildew species cannot be reliably distinguished based on their ITS sequences because these are sometimes identical (Takamatsu et al., 2015a), or the intra-specific, and intra-sample variation is sometimes higher than the differences between the ITS sequences reported for closely related taxa (Kovács et al., 2011; Ellingham et al., 2019).

Another fundamental bias of the molecular identification of powdery mildews is the lack of DNA sequence information from the type specimens of hundreds of species described and named several decades ago. All those species are currently identified by their morphologies and ITS sequences determined in fresh specimens collected from the respective host plant species. Epitypification (ICN, Art. 9.9) is an established and useful tool to overcome problems in the genetic characterization of ascomycete species, including powdery mildews, when type specimens cannot be used for genetic analyses due to their age, state of conservation, or sparseness. Furthermore, many plant species, and even single leaves of the same host plants, are sometimes infected with more than one powdery mildew species (Chen et al., 2008; Kiss et al., 2008; Krístková et al., 2009), and these could be morphologically indistinguishable (Takamatsu et al., 2002; Jankovics et al., 2008; Bereczky et al., 2015; Desprez-Loustau et al., 2018). Such mixed infections may complicate the identification of powdery mildews. In obligate biotrophs, the host range is decisive in speciation as growth and asexual and sexual reproduction of these pathogens take place while structurally and nutritionally linked to the living host tissues. Gene flow cannot happen between obligate biotrophic pathogens which do not share the same hosts. However, still little is known about the host ranges of morphologically similar powdery mildews that exhibit identical, or highly similar ITS sequences. To date, only a few experimental studies have determined the host ranges of these powdery mildews (Hirata et al., 2000; Jankovics et al., 2008; Pastirčáková et al., 2016). Although phylogenies based on DNA sequences should not be regarded as species phylogenies (Doyle, 1992), the current practice is to apply the same taxon names for powdery mildews that share identical, or highly similar ITS sequences (e.g., Takamatsu et al., 2015a,b; Braun et al., 2019). This approach has been widely applied even in the absence of experimental evidence for an overlap in the host plant ranges of the respective pathogens, i.e., whether powdery mildews with identical, or highly similar ITS sequences can actually meet and recombine in the field. Our study has followed this current practice in powdery mildew research, although we recognize that many more experimental host range studies are needed to reliably delimit closely related taxa based on their potential constraints in gene flow, i.e., what can reasonably be considered true biological species.

There are two powdery mildew taxa, namely *G. ixodiae* on the native *I. achillaeoides* (Cunnington et al., 2005a), and *G. lycopersici* infecting tomato (Kiss et al., 2001) that have never been identified outside Australia. Based on their morphologies and ITS sequences, both taxa are closely related to *G. longipes*, a well-known pathogen of solanaceous vegetables and ornamentals in Europe and the USA (Kiss et al., 2008; Kovács et al., 2011). Also, an *Euoidium* sp. recorded on *N. alata* and *S. melongena* in Australia has identical ITS sequences to those of *G. longipes* and *G. lycopersici* (Cunnington et al., 2005b). More work, including experimental host range studies are needed to determine whether all these taxa in fact represent the same globally distributed species.

*Neoerysiphe kerribeeensis* is the only taxon which has been described as a native Australian teleomorphic powdery mildew species producing chasmothecia on the native *S. glossanthus*. Its asexual morph was identified on several *Senecio* spp. introduced to Australia (Beilharz et al., 2010). There are no closely related *Neoerysiphe* species known overseas; therefore, the origin of *N. kerribeeensis* needs further investigation. Beilharz et al. (2010) suggested that *N. kerribeeensis* was native to Australia, and had become a pathogen of introduced *Senecio* spp. A contrasting explanation is that this species reached Australia as a pathogen of introduced *Senecio* spp., and its current occurrence on the native *S. glossanthus* is the result of its rapid host range expansion. If *N. kerribeeensis* was found overseas, this would support the second hypothesis.

Some powdery mildews become invasive when introduced to new geographic regions where their host plant species are also present (Kiss, 2005; Jones and Baker, 2007; Brasier, 2008; Desprez-Loustau et al., 2010, 2018). The introduction of *E. necator* from North America to Europe with infected grapevines is a textbook example (Brewer and Milgroom, 2010; Gadoury et al., 2012). Amongst all crop pathogenic fungi, a number of powdery mildews appear to be spreading most rapidly on a global scale, and are predicted to reach all available hosts by 2050 in many countries of the world (Bebber et al., 2014, 2019; Bebber and Gurr, 2015). Most probably, their introduction and spread in Australia has happened as part of the fundamental changes in the entire vegetation and landscape since 1788 (Kirkpatrick, 1999; Cook and Dias, 2006; Fensham and Laffineur, 2019). As conidia of powdery mildews are short-lived, and their long-distance dispersal is debated (Glawe, 2008), it is likely that all the species identified in this study had reached Australia on their living host plants.

Molecular clock studies indicated that the Erysiphales may have emerged in the late Cretaceous (~100–80 million years ago), and the first radiation of the major lineages occurred in the Cretaceous/Paleogene boundary (70–58 million years ago), in the Northern Hemisphere (Takamatsu, 2013a). By that time the Australian continent may have already been separated from other large pieces of land, so its terrestrial flora continued to evolve in isolation, without being exposed to those plant pathogenic fungi that could not reach the continent from overseas.

It is often difficult to explain how plant pathogenic fungi, especially plant-associated obligate biotrophs, reach new and remote geographic regions, particularly if these pathogens infect weeds and other wild plant species (Fontaine et al., 2013; Kiss et al., 2018b). The possibility that some powdery mildew species arrived in Australia prior to the first European settlement in 1788 cannot be excluded, particularly given Dutch exploration of the Australian continent from the early 1600s. Indigenous Australians arrived at least 50,000 years prior to the beginning of modern agriculture, and may have introduced a number of plant species to the Australian continent. Most of the taxa confirmed by this study are linked to the modern agricultural and horticultural trade, and recent, accidental, human-mediated introductions. The number of powdery mildew species in Australia compared to other parts of the world is low. The close phylogenetic relationships of all Australian powdery mildews to those known overseas, including the specimens collected from Australian native plants, indicate that Australia is a continent without native powdery mildews. Most, if not all, powdery mildew species in Australia appear to have been introduced since the European colonization of the continent.

## Data Availability Statement

The original contributions presented in the study are included in the article/Supplementary Material, further inquiries can be directed to the corresponding author/s (Vilgalys, 2003).

## Author Contributions

LKi designed and coordinated the research. LKi, NV, and UB wrote the paper. LKi, JD, ST, KB, JB, MC, AD, RGS, PD, LJ, TN, JE, WH, LKe, SM, KS, JM, SP, S-YL, RSm, RSo, UT, KV, AB, DW, and AY collected and examined the fresh specimens and determined some of the ITS sequences. CM, JD, and KB provided botanical expertise and UB and RGS taxonomic expertise. YT examined the old herbarium specimens and sequenced the ITS region in those specimens. LKi, KB, KS, LKe, D-NJ, and MN determined the rest of the nrDNA sequences. NV carried out the phylogenetic analyses. LKi and ST contributed to those analyses. All authors commented on the manuscript and read and approved the final version.

### Conflict of Interest

The authors declare that the research was conducted in the absence of any commercial or financial relationships that could be construed as a potential conflict of interest.
