# Supplementary material for: Australia: A Continent Without Native Powdery Mildews? The First Comprehensive Catalog Indicates Recent Introductions and Multiple Host Range Expansion Events, and Leads to the Re-discovery of Salmonomyces as a New Lineage of the Erysiphales
Source: Front Microbiol. 2020 Jul 16;11:1571. doi: 10.3389/fmicb.2020.01571 (PMC7378747; doi:10.3389/fmicb.2020.01571)
Supplement: Supplementary file 1 [file Data_Sheet_1.docx]

Supplementary Material

# Supplementary Figure


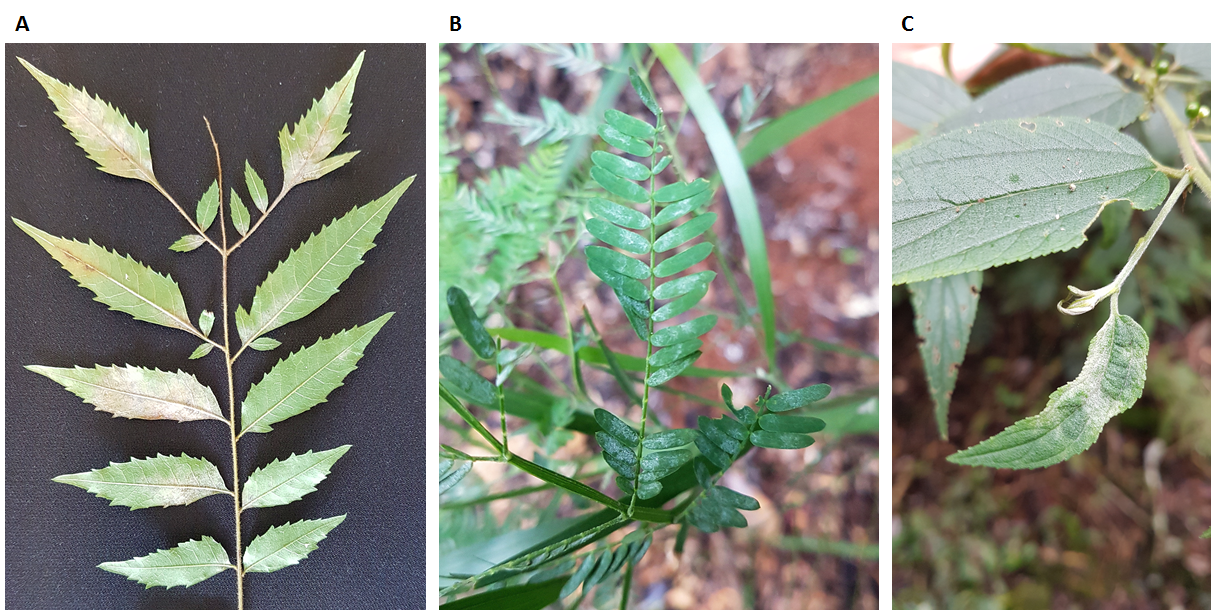


**Supplementary Figure 1.** Symptoms of powdery mildew infections on three Australian natives. **(A)** *Erysiphe quercicola* on the lower leaf surfaces of *Jagera pseudorhus* (BRIP 68798) **(B)** *Erysiphe* cf. *trifoliorum* on the bipinnate leaves of the tips of the phyllodes of *Acacia orites* (BRIP 70580) **(C)** *Podosphaera xanthii* on a young leaf of *Trema tomentosa* (BRIP 70495)

# Supplementary Tables 1-3

**Supplementary Table 1.** Powdery mildew specimens from *Acalypha* spp. collected in China and Argentina, and examined in this work.

| **Voucher specimen**^a^ | **Host plant species** | **Date of collection** | **Place of collection** |
| --- | --- | --- | --- |
| HMAS 153 | *Acalypha brachystachya* | Nov 1938 | Kunming, Yunnan Province, China |
| HMAS 152 | *A. brachystachya* | 9 Aug 1938 | Kunming, Yunnan Province, China |
| HMAS 155 | *A. brachystachya* | 26 Oct 1938 | Kunming, Yunnan Province, China |
| HMAS 1293 | *A. brachystachya* | 9 Aug 1938 | Kunming, Yunnan Province, China |
| HMAS 3603 | *A. brachystachya* | unknown | Kunming, Yunnan Province, China |
| HMAS 4328 | *A. brachystachya* | 9 Sept 1943 | Kunming, Yunnan Province, China |
| HMAS 40301 | *A. brachystachya* | 30 Aug 1959 | Dali, Yunnan Province, China |
| HAL 3297 F, HMJAU-PM91866 | *A. wilkesiana* | 25 April 2018 | Corrientes, Argentina |

^a^ HMAS: Herbarium of the Chinese Academy of Sciences, Beijing, China; HAL: Herbarium of Martin Luther University, Halle, Germany; HMJAU: Herbarium of Mycology of Jilin Agricultural University, Jilin, China.

**Supplementary Table 2.** Sequence data for powdery mildew specimens included in the phylogenetic analyses of the *Erysiphales* as representatives of different genera.

| **Powdery mildew species** | **Voucher specimen**^a^ | **Host plant species** | **GenBank accession no.**^b^ | | |
| --- | --- | --- | --- | --- | --- |
|  |  |  | **28S** | **18S** | **ITS** |
| *Salmonomyces acalyphae* | BRIP 68805 | *Acalypha nemorum* | MT133551 | MT133549 | MT133545 |
|  | BRIP 68804 | *A. wilkesiana* | MT133550 | MT133548 | MT133544 |
|  | MUMH5149 | *A. wilkesiana* | AB733594 | - | AB733589 |
|  | MUMH5150 | *A. wilkesiana* | AB733595 | - | AB733590 |
|  | MUMH5152 | *A. argentina* | AB733596 | - | AB733592 |
|  | MUMH5559 | *A. wilkesiana* | AB733597 | - | AB733593 |
| *Arthrocladiella mougeotii* | MUMH135 | *Lycium chinense* | AB022379 | AB033477 | AB022380 |
| *Blumeria graminis* | MUMH117 | *Bromus catharticus* | AB022362 | AB033475 | AB000935 |
| *Brasiliomyces malachrae* | MUMH3093 | *Malvastrum coromandelianum* | LC191217 | - | LC191217 |
| *Bulbomicroidium bauhiniicola* | MUMH6844 | *Bauhinia macranthera* | LC222311 | - | LC222311 |
| *Caespitotheca forestalis* | MUMH1461 | *Schinopsis balansae* | AB193467 | AB193465 | AB193466 |
| *Cystotheca wrightii* | MUMH137 | *Quercus glauca* | AB022355 | AB120747 | AB000932 |
| *Erysiphe adunca* | MUMH39 | *Salix vulpina* | AB022374 | AB022373 | D84383 |
| *E. aquilegiae* | MUMHS12 | *Cimicifuga simplex* | AB022405 | AB022404 | AB000944 |
| *E. australiana* | n.a.^c^ | *Lagerstroemia indica* | AB022407 | AB022406 | AB022408 |
| *E. cornicola* | MUMH90 | *Cornus controversa* | AB022389 | AB022388 | AB000941 |
| *E. friesii* | MUMH6 | *Rhamnus japonica* | AB022382 | AB033478 | AB000939 |
| *E. glycines* | MUMH52 | *Desmodium podocarpum* | AB022397 | AB120748 | AB015927 |
| *E. heraclei* | MUMH73 | *Daucus carota* | AB022391 | AB022390 | AB000942 |
| *E. hiratae* | MUMH112 | *Quercus glauca* | AB022357 | AB022356 | AB022358 |
| *E. japonica* | MUMHs76 | *Quercus cuspidata* | AB022415 | AB120752 | AB022416 |
| *E. mori* | MUMHs77 | *Morus australis* | AB022418 | AB033484 | AB000946 |
| *E. trina* | MUMH114 | *Quercus agrifolia* | AB022350 | LC456707 | AB022351 |
| *Golovinomyces circumfusus* | MUMH37 | *Eupatorium chinense* | AB022360 | AB022359 | AB000934 |
| *Leveillula taurica* | MUMH124 | *Capsicum annum* | AB022387 | AB033479 | AB000940 |
| *Microidium phyllanthi* | MUMH1782 | *Phyllanthus amarus* | AB120755 | AB120756 | LC259487 |
| *M. phyllanthi-reticulati* | MUMH1761 | *Phyllanthus reticulatus* | AB120758 | AB120757 | LC259486 |
| *Neoerysiphe galeopsidis* | MUMHS132 | *Chelonopsis moschata* | AB022369 | AB120749 | AB022370 |
| *Parauncinula polyspora* | MUMH197 | *Quercus cuspidata* | AB022420 | AB183531 | AB022421 |
| *P. septata* | MUMH585 | *Quercus* sp. | AB183532 | AB183530 | AB183533 |
| *Phyllactinia moricola* | MUMH35 | *Moris australis* | AB022401 | AB033481 | D84385 |
| *Pleochaeta shiraiana* | MUMH36 | *Celtis sinensis* | AB022403 | AB120750 | D84381 |
| *Podosphaera longiseta* | MUMH70 | *Prunus grayana* | AB022423 | AB120751 | AB000945 |
| *Po. xanthii* | MUMH68 | *Melothria japonica* | AB022410 | AB033482 | D84387 |
| *Queirozia turbinata* | VIC 26560 | *Platycyamus regnellii* | AB218773 | AB218773 | AB218773 |
| *Sawadaea polyfida* | MUMH47 | *Acer palmatum* | AB022364 | AB033476 | AB000936 |

^a^ BRIP: Queensland Plant Pathology Herbarium, Brisbane, Qld, Australia; MUMH: Mie University Mycological Herbarium, Tsu, Japan; VIC: Herbarium of the Universidade Federal de Vicosa, Brazil.

^b^ 28S: large subunit of the nuclear ribosomal DNA (nrDNA); 18S: small subunit of the nrDNA; ITS: the internal transcribed spacers and the intervening 5.8S region of the nrDNA.

^c^ Voucher specimen not available.

**Supplementary Table 3.** Powdery mildew specimens from overseas included in the plylogenetic analyses based on nrDNA ITS sequences.

| **Powdery mildew species** | **Host plant species** | **Herbarium accession number** | **Location** | **Year of collec-tion** | **ITS Genbank accession number** | **Reference** |  |
| --- | --- | --- | --- | --- | --- | --- | --- |
| *Erysiphe alphitoides* | *Quercus robur* | MUMH 773 | Lithuania | 1999 | AB292710 | Takamatsu et al. (2007) | |
|  | *Quercus dentata* | MUMH 2620 | Japan | 2003 | AB292701 | Takamatsu et al. (2007) | |
|  | *Quercus alba* | MUMH 3178 | Argentina | 2004 | AB292703 | Takamatsu et al. (2007) | |
| *E. aquilegiae* | *Clematis terniflora* | MUMH 0098 | Japan | 1994 | LC009920 | Takamatsu et al. (2015) | |
|  | *Aquilegia vulgaris* | MUMH 2456 | Argentina | 2004 | LC010016 | Takamatsu et al. (2015) | |
| *E. cruciferarum* | *Brassica rapa* | HMQAU 12216 | China | 2013 | KC878683 | Zhao et al. (2014) | |
|  | *Brassica juncea* | MUMH 7034 | Azerbaijan | 2016 | LC270859 | Abasova et al. (2018) | |
| *Erysiphe* cf. *trifoliorum* | *Vicia faba* | MUMH 837 | Japan | 1999 | AB079854 | Okamoto et al. (2002) | |
|  | *Vivia nigricans* | MUMH 2438 | Argentina | 2004 | LC010014 | Takamatsu et al. (2015) | |
| *E. diffusa* | *Wisteria sinensis* | HMJAU 02177 | China | 2013 | KM260363 | Fu et al. (2015) | |
|  | *Glycine max* | MUMH 1464 | USA | 1999 | AB078811 | Takamatsu et al. (2002) | |
|  | *Carica papaya* | VIC 26556 | Brazil | 2003 | LC228615 | Braun et al. (2017) | |
| *E. euonymicola* | *Euonymus fortunei* | KUS-F23304 | Korea | 2014 | KM361621 | Lee et al. (2015) | |
|  | *Euonymus japonicus* | MUMH 6999 | Azerbaijan | 2016 | LC270834 | Abasova et al. (2018) | |
| *E. guarinonii* | *Laburnum alpinum* | MUMH 1425 | Switzerland | 1999 | LC009983 | Takamatsu et al. (2015) | |
| *E. heraclei* | *Petroselinum crispum* | KUS-F25037 | Korea |  | KF680162 | Cho et al. (2014) | |
| *E. izuensis* | *Rhododendron macrosepalum* | MUMH 0535 | Japan | 1998 | LC009950 | Takamatsu et al. (2015) | |
|  | *Rhododendron oomurasaki* | MUMH 1131 | Japan | 2000 | LC009975 | Takamatsu et al. (2015) | |
| *E. palczewskii* | *Caragana arborescens* | MUMH 2581 | Ukraine | 1997 | LC010048 | Takamatsu et al. (2015) | |
| *E. pisi* | *Pisum sativum* | DNA03 | Japan |  | LC009890 | Takamatsu et al. (2015) | |
|  | *Pisum sativum* | OE2016PM CS81 | United Kingdom |  | KY653210 | Ellingham et al. (2019) | |
|  | *Pisum sativum* | n.a. |  |  | CACM 00000000 | Genbank | |
|  | *Lathyrus latifolius* | UC1512315 | USA |  | AF011306 | Saenz and Taylor (1999) | |
| *E. platani* | *Platanus orientalis* | KR29265 | Greece | 2011 | JQ365943 | Scholler et al. (2012) | |
| *E. quercicola* | *Quercus phillyraeoides* | MUMH 885 | Japan | 1999 | AB193591 | Limkaisang et al. (2005) | |
| *E. sedi* | *Sedum pallescens* | MUMH 2577 | Russia | 1999 | LC010047 | Takamatsu et al. (2015) | |
| *E. syringae* | *Syringa vulgaris* | MUMH 1032 | USA | 1999 | AB295460 | Seko et al. (2011) | |
| *Erysiphe* sp. | *Vicia faba* | GUM779 | Iran | 2012 | MF663774 | Khodaparast et al. (2016) | |
| *E. trifoliorum* | *Trifolium arvense* | MUMH 701 | Hungary | 1999 | LC009955 | Takamatsu et al. (2015) | |
|  | *Trifolium pratense* | MUMH 1046 | USA | 1999 | LC009972 | Takamatsu et al. (2015) | |
|  | *Medicago littoralis* | MUMH 7038 | Azerbaijan | 2016 | LC270860 | Abasova et al. (2018) | |
| *Golovinomyces latisporus* | *Helianthus tuberosus* | ERY061 | Russia | 2018 | MK452644 | Qiu et al. (2020) | |
|  | *Helianthus annuus* | HMJAU-PM91831 | China | 2018 | MK452602 | Qiu et al. (2020) | |
|  | *Zinnia angustifolia* | HAL 2338 F | Germany | 2008 | MK452631 | Qiu et al. (2020) | |
|  | *Helianthus annuus* | LM0P03825217-2 | USA | 2018 | MK452636 | Qiu et al. (2020) | |
|  | *Helianthus annuus* | HAL 3299 F | Switzerland | 2018 | MK452627 | Qiu et al. (2020) | |
| *G. asterum* var. *solidaginis* | *Solidago altissima* | MUMH 54 | Japan | 1994 | AB077625 | Matsuda & Takamatsu (2003) | |
| *G. biocellatus* | *Melissa officinalis* | HAL 2369F | Hungary | 2010 | HM156493 | Kassai-Jáger et al. (2010) | |
|  | *Agastache rugosa* | HMJAU 02286 | China |  | KT991148 | GenBank | |
|  | *Lycopus europaeus* | KR M 35027 | Germany | 2012 | LC076832 | Scholler et al. (2016) | |
| *G. bolayi* | *Papaver somniferum* | MUMH 1037 | USA | 1999 | AB769463 | Takamatsu et al. (2013) | |
|  | *Arabidopsis thaliana* | MUMH 2355 | Hungary | 2003 | AB769457 | Takamatsu et al. (2013) | |
| *G. glandulariae* | *Verbena hastata* | KR M 43410 | Germany | 2003 | LC076839 | Scholler et al. (2016) | |
|  | *Verbena* sp. | KR M 43411 | Germany | 2002 | LC076840 | Scholler et al. (2016) | |
| *G. longipes* | *Petunia x hybrida* | BPI 878251 | USA | 2006 | EU327321 | Kiss et al. (2008) | |
|  | *Petunia x hybrida* | BPI 878253 | Hungary | 2006 | EU327322 | Kiss et al. (2008) | |
|  | *Torenia furnieri* | HAL 1922F | Hungary |  | DQ538345 | Vági et al. (2007) | |
| *G. orontii* | *Capsella bursa-pastoris* | IHPS F50 | Slovenia | 2017 | MG952279 | Radisek et al. (2018a) | |
|  | *Viola arvensis* | MUMH 1406 | Switzerland | 1996 | AB769472 | Takamatsu et al. (2013) | |
|  |  |  |  |  |  |  | |
| *Leveillula lactucarum* | *Lactuca orientalis* | MUMH 4883 | Iran | 2006 | AB667860 | Khodaparast et al. (2012) | |
|  | *Lactuca* sp. | MUMH 4900 | Iran | 2007 | AB667861 | Khodaparast et al. (2012) | |
| *L. taurica* | *Carthamus* sp. | MUMH 1421 | Iran | 1999 | AB667864 | Khodaparast et al. (2012) | |
|  | *Cartharanthus roseus* | BPI 892677 | USA | 2013 | KF703447 | Romberg et al. (2014) | |
|  | *Cucurbita ficifolia* | KUS-F28536 | Korea | 2014 | MH698492 | Choi et al. (2019) | |
|  | *Echinops pungens* | MUMH 4901 | Iran | 2004 | AB667871 | Khodaparast et al. (2012) | |
|  | *Eryngium* sp. | MUMH 4885 | Iran | 2006 | AB667872 | Khodaparast et al. (2012) | |
|  | *Euphorbia petiolata* | n.a. | Iran |  | AB045004 | Khodaparast et al. (2012) | |
|  | *Hyoscyamus* sp. | MUMH4892 | Iran | 2006 | AB667876 | Khodaparast et al. (2012) | |
|  | *Ononis spinosa* | MUMH 4888 | Iran | 1997 | AB667876 | Khodaparast et al. (2012) | |
|  | *Plumbago europaea* | MUMH 4227 | Iran | 1948 | Ab667880 | Khodaparast et al. (2012) | |
|  | *Rosa* sp. | MUMH 4897 | Iran |  | AB667881 | Khodaparast et al. (2012) | |
|  | *Sanguisorba* sp. | MUMH 4899 | Iran | 2003 | AB667882 | Khodaparast et al. (2012) | |
|  | *Vicia* sp. | MUMH 4223 | Iran | 2006 | AB667884 | Khodaparast et al. (2012) | |
| *Microidium phyllanthi* | *Phyllantus acidus* | MUMH 3361 | Thailand | 2004 | AB719943 | Meeboon & Takamatsu (2017) | |
|  | *Phyllantus urinaria* | HMJAU 91783 | China |  | MH359093 | GenBank | |
| *Podosphaera aphanis* | *Fragaria* x *ananassa* | HMJAU 02284 | China | 2015 | KT359262 | GenBank | |
|  | *Fragaria chiloensis* | MUMH 1871 | Argentina | 2001 | AB525933 | Takamatsu et al. (2010) | |
| *P. leucotricha* | *Pyrus calleryana* | BPI 878262 | Hungary | 2007 | EU148597 | Vajna & Kiss (2008) | |
|  | *Prunus persica* | BPI 880514 | Serbia | 2008 | HM579839 | Jankovics et al. (2011) | |
|  | *Malus domestica* | OE2015PM13CS | United Kingdom |  | KY661017 | Ellingham et al. (2019) | |
| *P. pannosa* | *Rosa* sp. | CMPH-105 | Mexico | 2012 | KF753690 | GenBank | |
|  | *Prunus persica* | HAL 2386F | France | 2008 | HM579843 | Jankovics et al. (2011) | |
| *P. plantaginis* | *Plantago lanceolata* | OE2016PMCS44 | United Kingdom |  | KY661120 | Ellingham et al. (2019) | |
|  | *Plantago lanceolata* | PDD 105904 | New Zealand | 2015 | MK432775 | GenBank | |
| *P. tridactyla* | *Prunus padus* | VPRI 20233 | South Korea |  | AY833652 | Cunnington et al. (2005) | |
|  | *Prunus laurocerasus* | VPRI 22157 | Switzerland |  | AY833654 | Cunnington et al. (2005) | |
| *P. xanthii* | *Petunia x hybrida* | BPI 878254 | USA | 2006 | EU327326 | Kiss et al. (2008) | |
|  | *Matricaria chamomilla* | GUM781 | Iran | 2011 | MF663781 | Khodaparast (2016) | |
|  | *Hydrocleys nymphoides* | KUS F27719 | Korea | 2013 | MF402945 | Cho et al. (2018) | |
|  | *Impatiens textori* | MUMH 245 | Japan | 1996 | AB040344 | Hirata et al. (2000) | |
| *Pseudoidium hortensiae* | *Hydrangea macrophylla* | KUS-F25514 | Korea | 2011 | JQ669944 | Park et al. (2012) | |
|  | *Hydrangea macrophylla* | MUMH 0071 | Japan | 1994 | LC009915 | Takamatsu et al. (2015) | |
| *Pseudoidium* sp. | *Saxifraga stolonifera* | MUMH 4037 | Japan | 2005 | LC010064 | Takamatsu et al. (2015) | |
|  | *Solanum betaceum* | MUMH 4922 | India | 2008 | AB473221 | Baiswar et al. (2013) | |
|  | *Lathyrus magellanicus* | MUMH 2442 | Argentina | 2004 | LC010015 | Takamatsu et al. (2015) | |
| *Sawadaea bicornis* | *Acer pseudoplatanus* | MUMH 904 | United Kingdom | 1999 | AB193380 | Hirose et al. (2005) | |
|  | *Acer campestre* | MUMH 1062 | Armenia | 1981 | AB193379 | Hirose et al. (2005) | |
| *S. polyfida* | *Acer amoenum* var. *matsumurae* | MUMH 486 | Japan | 1998 | AB193358 | Hirose et al. (2005) | |
|  | *Acer palmatum* | MUMH 551 | Japan | 1998 | AB193382 | Hirose et al. (2005) | |

**REFERENCES for Suppl. Table 3:**

Abasova, L.V., Aghayeva, D.N., &Takamatsu, S. (2018) Notes on powdery mildews of the genus *Erysiphe* from Azerbaijan. *Current Research in Environmental & Applied Mycology* 8: 30-53.

Baiswar, P., Ngachan, S. V., Braun, U., Takamatsu, S., Chandra, S., & Harada, M. (2013) Molecular characterization of *Oidium* sp. on *Solanum betaceum* in India. *Environment & Ecology* 31: 1364-1367.

Braun, U., Meeboon, J., Takamatsu, S., Blomquist, C., Fernandez Pavia, S. P., Rooney-Latham, S., & Macedo, D. M. (2017) Powdery mildew species on papaya – a story of confusion and hidden diversity. *Mycosphere* 8: 1403-1423.

Cho, S. E., Han, K. S., Choi, I. Y., & Shin, H. D. (2018) First report of powdery mildew caused by *Podosphaera xanthii* on *Hydrocleys nymphoides* in Korea. *Plant Disease* 102: 247.

Cho, S. E., Park, M. J., Park, J. H., Kim, J. Y., & Shin, H. D. (2014) First report of powdery mildew caused by Erysiphe heraclei on Parsley in Korea. *Plant Disease* 98: 847.

Choi, I. Y., Jang, S. J., Oh, H. T., & Shin, H. D. (2019) First report of powdery mildew caused by *Leveillula taurica* on *Cucurbita ficifolia* in Korea. *Plant Disease* 103: 586.

Ellingham, O., David, J., & Culham, A. (2019) Enhancing identification accuracy for powdery mildews using previously underexploited DNA loci. *Mycologia* 111: 798-812.

Fu, X. Y., Liu, S. Y., Jiang, W. T., & Li, Y. (2015) *Erysiphe diffusa*: a newly recognized powdery mildew pathogen of *Wisteria sinensis*. *Plant Disease* 99: 1272.

Hirata, T., Cunnington, J. H., Paksiri, U., Limkaisang, S., Shishkoff, N., Grigaliunaite, B., Sato, Y., & Takamatsu, S. (2000) Evolutionary analysis of subsection *Magnicellulatae* of *Podosphaera* section *Sphaerotheca* (Erysiphales) based on the rDNA internal transcribed spacer sequences with special reference to host plants. *Canadian Journal of Botany* 78: 1521-1530.

Hirose, S., Tanda, S., Levente, K. I. S. S., Grigaliunaite, B., Havrylenko, M., & Takamatsu, S. (2005) Molecular phylogeny and evolution of the maple powdery mildew (*Sawadaea*, Erysiphaceae) inferred from nuclear rDNA sequences. *Mycological Research* 109: 912-922.

Jankovics, T., Dolovac, N., Bulajić, A., Krstić, B., Pascal, T., Bardin, M., Nicot, P. C., & Kiss, L. (2011) Peach rusty spot is caused by the apple powdery mildew fungus, *Podosphaera leucotricha*. *Plant Disease* 95: 719-724.

Kabaktepe, S., Akata, I., Siahaan, S. A., Takamatsu, S., & Braun, U. (2017) Powdery mildews (Ascomycota, Erysiphales) on *Fontanesia phillyreoides* and *Jasminum fruticans* in Turkey. *Mycoscience* 58: 30-34.

Kassai-Jáger, E., Kiss, L., Vaczy, Z., & Vaczy, K. Z. (2010) First report of powdery mildew on lemon balm (*Melissa officinalis*) caused by *Golovinomyces biocellatus* in Hungary. *Plant Disease* 94: 1169.

Khodaparast, S. A. (2016) Molecular identification of some anamorphic powdery mildews (Erysiphales) in Guilan province, north of Iran. *Mycologia Iranica* 3: 127-133.

Khodaparast, S. A., Takamatsu, S., Harada, M., Abbasi, M., & Samadi, S. (2012) Additional rDNA ITS sequences and its phylogenetic consequences for the genus *Leveillula* with emphasis on conidium morphology. *Mycological Progress* 11: 741-752.

Kiss, L., Jankovics, T., Kovács, G. M., & Daughtrey, M. L. (2008) *Oidium longipes*, a new powdery mildew fungus on petunia in the USA: a potential threat to ornamental and vegetable solanaceous crops. *Plant Disease* 92: 818-825.

Lee, C. K., Lee, S. K., Lee, S. H., Cho, S. E., & Shin, H. D. (2015) First report of powdery mildew caused by *Erysiphe euonymicola* on *Euonymus fortunei* var. *radicans* in Korea. *Plant Disease* 99: 556.

Limkaisang, S., Kom-un, S., Furtado, E. L., Liew, K. W., Salleh, B., Sato, Y., & Takamatsu, S. (2005) Molecular phylogenetic and morphological analyses of *Oidium heveae*, a powdery mildew of rubber tree. *Mycoscience* 46: 220-226.

Matsuda, S., & Takamatsu, S. (2003) Evolution of host–parasite relationships of *Golovinomyces* (Ascomycete: Erysiphaceae) inferred from nuclear rDNA sequences. *Molecular Phylogenetics and Evolution* 27: 314-327.

Meeboon, J., & Takamatsu, S. (2017) *Microidium phyllanthi-reticulati* sp. nov. on *Phyllanthus reticulatus*. *Mycotaxon* 132: 289-297.

Okamoto, J., Limkaisang, S., Nojima, H., & Takamatsu, S. (2002) Powdery mildew of prairie gentian: characteristics, molecular phylogeny and pathogenicity. *Journal of General Plant Pathology* 68: 200-207.

Park, M. J., Cho, S. E., Park, J. H., Lee, S. K., & Shin, H. D. (2012) First report of powdery mildew caused by *Oidium hortensiae* on mophead hydrangea in Korea. *Plant Disease* 96: 1072.

Park, M. J., Kim, B. S., Choi, I. Y., Cho, S. E., & Shin, H. D. (2015) First report of powdery mildew caused by *Golovinomyces ambrosiae* on sunflower in Korea. *Plant Disease* 99: 557.

Radisek, S., Jakse, J., Zhao, T. T., Cho, S. E., & Shin, H. D. (2018a) First report of powdery mildew of *Capsella bursa-pastoris* caused by *Golovinomyces orontii* in Slovenia. *Journal of Plant Pathology* 100: 359.

Radisek, S., Jakse, J., Zhao, T. T., Cho, S. E., & Shin, H. D. (2018b) First report of powdery mildew of *Helianthus tuberosus* caused by *Golovinomyces ambrosiae* in Slovenia. *Journal of Plant Pathology* 100: 331.

Romberg, M. K., Kennedy, A. H., & Ko, M. (2014) First report of the powdery mildews *Leveillula taurica* and *Podosphaera pannosa* on rose periwinkle in the United States. *Plant Disease* 98: 848.

Saenz, G. S., & Taylor, J. W. (1999) Phylogeny of the Erysiphales (powdery mildews) inferred from internal transcribed spacer ribosomal DNA sequences. *Canadian Journal of Botany* 77: 150-168.

Scholler, M, Hemm, V, & Lutz, M. (2012) *Erysiphe platani*: monitoring of an epidemic spread in Germany and molecular characterization based on rDNA sequence data. *Andrias* 19: 263-272.

Scholler, M., Schmidt, A., Siahaan, S. A. S., Takamatsu, S., & Braun, U. (2016) A taxonomic and phylogenetic study of the *Golovinomyces biocellatus* complex (Erysiphales, Ascomycota) using asexual state morphology and rDNA sequence data. *Mycological Progress* 15: 56.

Seko, Y., Bolay, A., Kiss, L., Heluta, V., Grigaliunaite, B., & Takamatsu, S. (2008) Molecular evidence in support of recent migration of a powdery mildew fungus on *Syringa* spp. into Europe from East Asia. *Plant Pathology* 57: 243-250.

Takamatsu, S., Braun, U., Limkaisang, S., Kom-Un, S., Sato, Y., & Cunnington, J. H. (2007) Phylogeny and taxonomy of the oak powdery mildew *Erysiphe alphitoides* sensu lato. *Mycological Research* 111: 809-826.

Takamatsu, S., Ito, H., Shiroya, Y., Kiss, L., & Heluta, V. (2015) First comprehensive phylogenetic analysis of the genus *Erysiphe* (Erysiphales, Erysiphaceae) I. The *Microsphaera* lineage. *Mycologia* 107: 475-489.

Takamatsu, S., Matsuda, S., & Grigaliunaite, B. (2013) Comprehensive phylogenetic analysis of the genus *Golovinomyces* (Ascomycota: Erysiphales) reveals close evolutionary relationships with its host plants. *Mycologia* 105: 1135-1152.

Takamatsu, S., Niinomi, S., Harada, M., & Havrylenko, M. (2010) Molecular phylogenetic analyses reveal a close evolutionary relationship between *Podosphaera* (Erysiphales: Erysiphaceae) and its rosaceous hosts. *Persoonia: Molecular Phylogeny and Evolution of Fungi* 24: 38.

Takamatsu, S., Taguchi, Y., Shin, H. D., Paksiri, U., Limkaisang, S., Binh, N. T., & Sato, Y. (2002) Two *Erysiphe* species associated with recent outbreak of soybean powdery mildew: results of molecular phylogenetic analysis based on nuclear rDNA sequences. *Mycoscience* 43: 333-341.

Vági, P., Kovacs, G. M., & Kiss, L. (2007) Host range expansion in a powdery mildew fungus (*Golovinomyces* sp.) infecting Arabidopsis thaliana: *Torenia fournieri* as a new host. *European Journal of Plant Pathology* 117: 89-93.

Vajna, L., & Kiss, L. (2008) First report of powdery mildew on *Pyrus calleryana* caused by *Podosphaera leucotricha*. *Plant Disease* 92: 176.

Zhao, H. H., Xing, H. H., Liang, C., Yang, X. Y., Cho, S. E., & Shin, H. D. (2014) First report of powdery mildew caused by *Erysiphe cruciferarum* on Chinese cabbage in China. *Plant Disease* 98: 421.
